# Basal Plane Doping to Activate Colloidal MoS_2_ Nanosheets for Catalytic Hydrodeoxygenation of *para-*Cresol

**DOI:** 10.1021/acsami.5c24853

**Published:** 2026-04-04

**Authors:** Steven L. Farrell, Noah Osinski, Ingrid J. Paredes, Amani M. Ebrahim, Haoxian Zheng, Yuchen Zhang, Christopher Oyuela, Lu Ma, Steven N. Ehrlich, Srinivas Rangarajan, Anatoly I. Frenkel, Ayaskanta Sahu

**Affiliations:** † Department of Chemical and Biomolecular Engineering, 5894 New York University, Brooklyn, New York 11201, United States; ‡ Department of Materials Science and Chemical Engineering, Stony Brook University, Stony Brook New York 11794, United States; § Department of Chemical and Biomolecular Engineering, 1687 Lehigh University, Bethlehem, Pennsylvania 18015, United States; ∥ National Synchrotron Light Source II, Brookhaven National Laboratory, New York, New York 11973, United States; ⊥ Chemistry Division, Brookhaven National Laboratory, Upton, New York 11973, United States

**Keywords:** structure−activity relationship, hydrodeoxygenation, molybdenum disulfide, doping, X-ray absorption spectroscopy, extended X-ray absorption fine structure

## Abstract

The valorization of biomass into biofuels is a critical process for producing renewable fuels. Hydrodeoxygenation (HDO), particularly over doped molybdenum disulfide (MoS_2_), a transition metal dichalcogenide (TMD) material, is a common representative catalytic reaction system for converting biomass-derived materials into useful hydrocarbons. However, the location and role of dopants, such as Co, in HDO is not fully understood. The effects of dopant location and oxidation state are often precluded by inhomogeneity in the ensemble properties of nanosheet size and dopant dispersion, as well as difficulty in observing the behavior of atomic site behavior directly. Using a colloidal approach to synthesize cobalt-doped MoS_2_ nanosheets with controlled dopant concentration, combined with X-ray absorption spectroscopy (XAS) and density functional theory (DFT) calculations, we determine that basal plane doped Co (25% Co:Mo mole ratio) shows peak catalytic activity in HDO of *para-*cresol, a model biomass-derived compound, and that basal Co sites are demonstrably more active than edge sites. By observing these doping effects in MoS_2_ catalysts for HDO, we can further optimize not only the production of carbon-neutral fuels but also direct the tailoring of doped TMD catalysts toward their intended applications.

## Graphical abstract

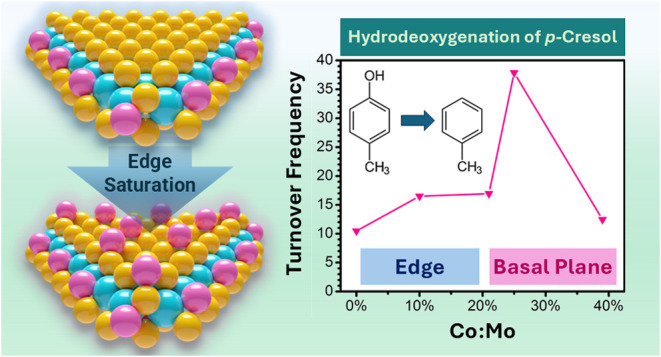

## Introduction

Upgrading biomass into biofuels has been a focus of efforts to provide and a secure and renewable energy source. The conversion of biomass into biofuels is typically achieved by a process known as hydrodeoxygenation (HDO), in which oxygen atoms are removed from lignin-derived molecules to produce energy-dense hydrocarbons and water.^1,2^ HDO requires intensive conditions to achieve the complete removal of oxygen.^3^ The impetus is to therefore develop catalysts that can efficiently and cost-effectively convert oxygen-rich feedstocks into valuable hydrocarbons at scale. Lignin and other biomass-derived materials pose a challenge in processing, due to their large size, complexity, and variation by source.^4,5^ Although lignin can be pyrolyzed into simpler, more manageable units, the resulting compounds still present a variety of different oxygen locations and functional groups.^6^ To address the complexity and difficulty of these reactions, it is vital for catalytic performance to be optimized for the efficient and selective cleavage of the C–O bonds.

While precious metal-based catalysts, such as platinum, are generally strong candidates for hydrogenation reactions, their susceptibility to poisoning by naturally occurring sulfur contaminants in biomass makes them a poor choice for HDO.^7,8^ Even without the risk of sulfur contamination, selectivity is difficult to control; in the case of precious metals, facilitated absorption of the aromatic ring may lead to ring hydrogenation before deoxygenation, consuming more hydrogen and energy than deoxygenation alone.^3,9^ In cases where high selectivity is required, such as the production of toluene or other aromatic compounds, balancing hydrogenation of the aromatic ring and the alcohol group is critical as both are possible pathways even for nonprecious metal catalysts.^10^ Ring hydrogenation is not only an inefficient use of energy on already deoxygenated products but also lowers the selectivity toward the target product.^9,11^ Molybdenum disulfide (MoS_2_) is a desirable alternative catalyst used for hydrogenation reactions, particularly in applications where sulfur is present, due to its sulfur poisoning resistance, low cost, stellar activity, and selectivity among nonprecious metal catalysts.^12–14^ MoS_2_ is a member of the transition metal dichalcogenides, a class of two-dimensional (2D) materials with layers held together in the *z*-direction by van der Waals forces and which can exist as stable single-layer sheets in the nanoscale. While MoS_2_ exhibits effective catalytic behavior, its activity is restricted to the edge sites; the basal plane is completely inert.^13,15^ Thus, the basal plane has been a target for activation in order to improve catalytic surface area, using methods such as vacancy formation and dispersed metal dopants.^16,17^ Cobalt- and nickel-based compounds have regularly been explored to great effect in converting lignin-derived compounds into hydrocarbons for fuels.^6,13,18^ Cobalt-doped MoS_2_ (Co-MoS_2_), for example, is often used in hydrogenation reactions, including HDO and hydrogen evolution.^19,20^ Besides HDO, Co-doped Mo catalysts, including Co-MoS_2_, have a history of effective use in sulfur and nitrogen oxides pollution prevention via hydrodesulfurization (HDS) and hydrodenitrogenation (HDN) of crude fuel feedstocks.^21–23^

While Co effectively improves the activity of MoS_2_ in hydrogenolysis reactions,^24–26^ the nature of the Co dopants is not well understood, namely the relationship between their local structure and the subsequent catalytic activity. In a previous report,^27^ we have explored a methodology for studying the structure–activity relationship present in colloidally synthesized nanoscale Co-MoS_2_ for HDS. In this case, Co was found to first attach to the edges of MoS_2_ before reaching a saturation point (∼16% Co:Mo, mol basis).^27^ Beyond edge saturation, Co begins to decorate the basal plane, unlocking the peak activity of these catalysts in HDS. Understanding the translation of this methodology is critical to understanding how other reactions, such as HDO can be optimized. However, the mechanisms governing the removal of sulfur heteroatoms over Co-MoS_2_ differ from those of oxygen. It is therefore important, in the interest of developing the design rules governing doped TMD catalysts, to apply this methodology to other hydrogenolysis reactions such as HDO and understand their similarities and differences toward catalyst optimization. While there is a healthy abundance of work on doping MoS_2_ for deoxygenating biomass-derived compounds, there is little work on understanding the nature of the structure–activity relationship for this system.

In this work, we employ robust, synchrotron-based X-ray characterization techniques, density functional theory (DFT) computation, and experimental catalysis studies to explore how Co location affects the HDO activity of Co-MoS_2_, as a methodology for probing the structure–activity relationship in dispersed-dopant MoS_2_ catalysts with a precision that cannot be achieved by other synthesis-probe combinations. Using colloidal, *in situ* doped nanosheets with tight control over dopant location and particle size, we investigate the selective conversion of *para-*cresol, a model biomass-derived compound, to toluene on edge and basal Co sites in doped MoS_2_. By observing the structure–activity relationship by this method, we can not only determine the effect of dopant local structure for improved biofuel valorization catalysis but also compare different hydrogenolysis reactions and better highlight mechanisms for designing future TMD catalysts tailored to their application.

## Results and Discussion

### Catalytic Activity in Hydrodeoxygenation

Catalysts were produced using a hot-injection colloidal synthesis approach, which allows for consistent, repeatable synthesis of nanosheets with similar sizes and tight size distribution, regardless of dopant inclusion.^28^ Prior to use, the Co:Mo mol ratios of synthesized catalysts were measured using inductively coupled plasma—mass spectrometry (ICP-MS). The resulting Co:Mo mol ratios ranged between 0% and 39% (0.39 mol Co:mol Mo). HDO of *p-*cresol was performed by combining each catalyst with a mixture of *p-*cresol, *n*-decane (reference compound) and tetralin. The conversion of *p-*cresol can follow multiple pathways, typically either through direct deoxygenation toward the desired product (toluene) or through hydrogenation to undesired intermediates and by products (4-methylcyclohexanol, methylcyclohexane); a reaction scheme can be found in the Supporting Information (Figure S1, Supporting Information). The nonpolar tetralin solvent served the function of dispersing the ligand-capped nanosheets; although tetralin has been reported as a hydrogen donor solvent for hydrogenolysis,^29,30^ we did not detect any significant formation of naphthalene that would be expected by hydrogen donation. In test reactions with tetralin solvent and no catalyst, no conversion of *p-*cresol was detected (Figure S2, Supporting Information). Figure 1a demonstrates the catalytic performance of Co-MoS_2_ nanosheets with various Co:Mo mol ratios as a function of reaction time. The inclusion of Co showed a definitive improvement over the bare MoS_2_ nanosheets, with 25% Co:Mo catalysts showing nearly twice the *p-*cresol conversion of the next best-performing Co concentration, 21% Co:Mo. Reaction rate equation fits (Figure 1b) and computed rate constants (Figure 1c) show that the reaction is of pseudo-first order nature with respect to *p-*cresol, given the large hydrogen excess. The rate constants of the reaction are calculated in Figure 1c. The optimal concentrations typically reported in literature widely vary, usually within a range of Co:Mo atomic ratio between 16 and 30% for the best performance, with which our data agree.^31–33^ However, it should be noted that these ratios are typically based on Co nanoparticles and clusters rather than single atoms, and with varied nanosheet morphologies. Morphology variation of MoS_2_ with similar Co loadings has also been shown to have a dramatic impact on *p-*cresol HDO activity.^26^ Additionally, the size of these sheets and the presence of ligands, particularly at high Co concentrations, may also play a role in activity and the effects governing design in the reported activities and this work may differ. The impact of size played a role in the activity as well; nanoscale MoS_2_ showed greatly improved *p*-cresol conversion over bulk MoS_2_ by a factor of 17.5 (Figure S2). Repeatability tests of 21% and 25% Co:Mo showed that this significant increase in activity was consistent, with standard deviation less than 2% for each after three tests. Recycle tests, where catalyst was recovered and reused over multiple runs, shows the catalyst was stable and reusable for multiple runs (Figure S3, Supporting Information).

**Figure 1:**
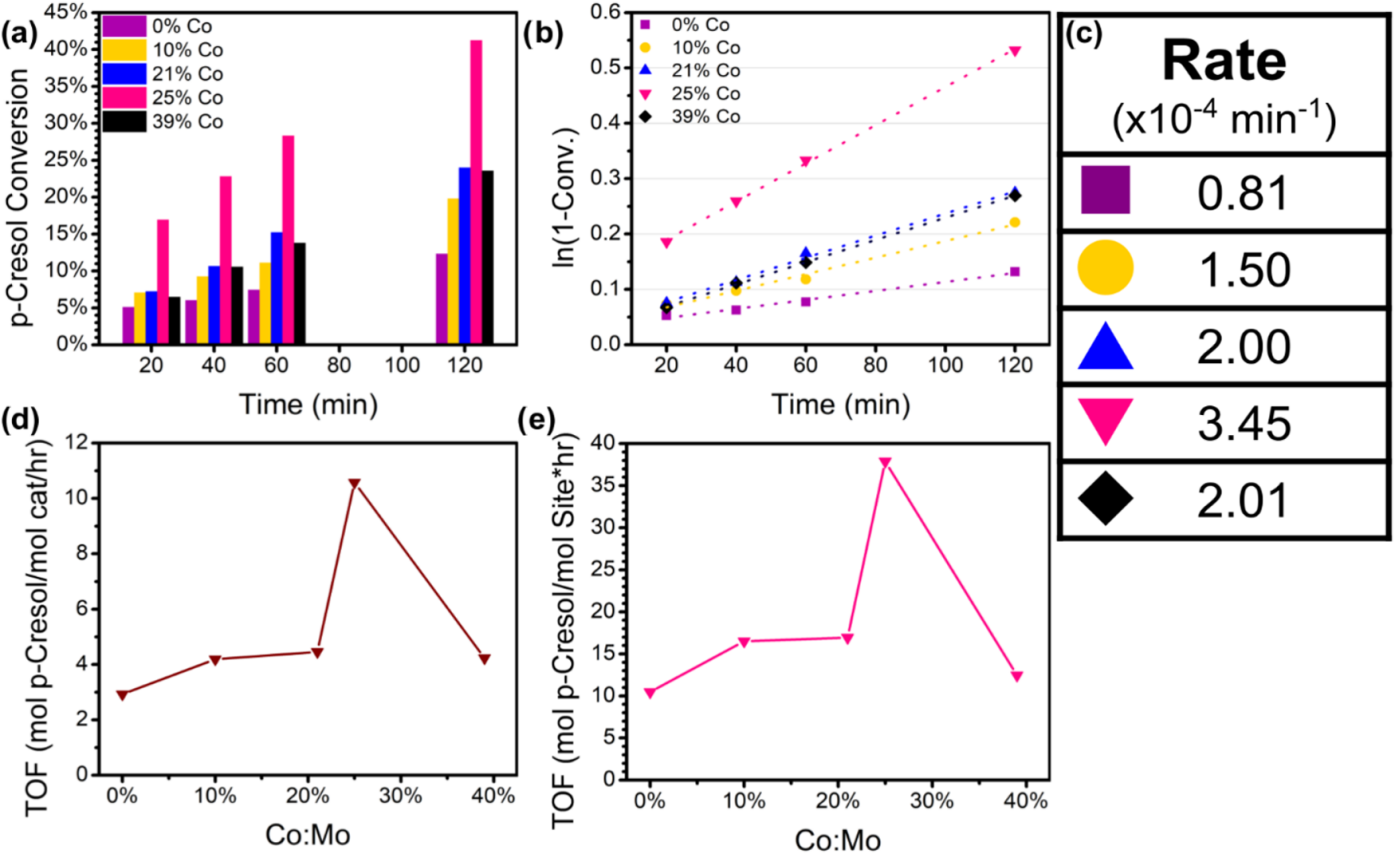
Hydrodeoxygenation studies of Co-MoS_2_ catalysts: (a) *p-*cresol conversion over 2 h using catalysts of various Co:Mo mol ratios, (b) resulting first-order reaction rate kinetics of Co-MoS_2_ nanosheets (dotted lines represent the line of best fit), (c) table of first order rate constants calculated from 1b, (d) turnover frequency - total catalyst mole basis, (e) turnover frequency - mol sites basis. Solid lines in 1d and 1e provide a visual guide showing the nonmonotonous trends in sample activity by varying Co:Mo mol ratio. 25% Co:Mo catalysts exhibited the highest performance in conversion of *p-*cresol with a 72.5% increase in reaction rate over the next highest catalyst at 21% Co:Mo.

The trend continues in the turnover frequency (TOF) per Co-MoS_2_ mole basis (Figure 1d) and per calculated mole site basis (Figure 1e). The mole site basis is calculated based on the average number of active sites, derived in a previous report (see SI for details).^27^ We also address the activity difference between Co-doped samples and the undoped nanoscale MoS_2_, rather than the activity itself, normalized to their respective Co concentrations to compare the impact of Co more accurately between samples (Figure S4, Supporting Information). This eliminates the influence of undoped edge sites of MoS_2_ that may still be present and unaccounted for at low Co concentrations, particularly at 10% Co:Mo (see SI for data and calculation details). This provided the relative improvement per unit Co. As we previously reported for this catalyst in HDS,^27^ there is a strong relationship between location and activity in HDO; in fact, the difference is much more dramatic, as the range for basal doping shows far greater HDO activity overall and per unit Co. Determining the source of disparity and the reasons for such improvement in HDO require further characterization of both the bulk structure and the local dopant structure.

### Structure of Co-MoS_2_

Typically, fresh MoS_2_ exists in a stable, semiconducting hexagonal structure, often denoted as the 2H phase (2H-MoS_2_). The synthesis of MoS_2_ nanosheets in an oleylamine environment as described instead produces nanosheets in a metastable, distorted trigonal structure, denoted as 1T’-MoS_2_.^27^ This phase is noted for its metallic behavior and active basal plane, making it attractive for catalytic applications.^16^ However, due to their metastability, the as-synthesized 1T’ nanosheets converted to the more stable 2H phase rapidly upon exposure to reaction conditions in hydrodesulfurization. We therefore employed various characterization techniques to determine if the same structural transition occurred during HDO, and found that the same transition to the 2H phase occurred. Figure 2a shows X-ray diffraction (XRD) performed on catalysts postuse in HDO. Due to their ultrathin nature, the XRD patterns for these nanosheets are broad. This stems from the Scherrer broadening effect, which has been observed in other reports of nanoscale MoS_2_ syntheses.^34–36^ The only minor appearance of the (002) peak, which would indicate stacking of multiple layers of MoS_2_, suggests that the catalysts maintained their nanoscale regime. The inclusion of Co makes minimal changes to the XRD and we cannot identify any specific Co-based peaks forming to indicate the presence of Co or Co oxide nanoparticles. XRD of fresh materials can be found in the SI (Figure S5). HRTEM images in 2b further support the nanoscale retention of MoS_2_ postuse in HDO, with nanosheets appearing as dark-lines due to static edge-anchoring effects on the TEM grid surface. A population count of 160 nanosheets (measured in Figures S6–S7 in the SI), reveal an average size of 4.21 ± 0.84 nm, as indicated in 2c.

**Figure 2:**
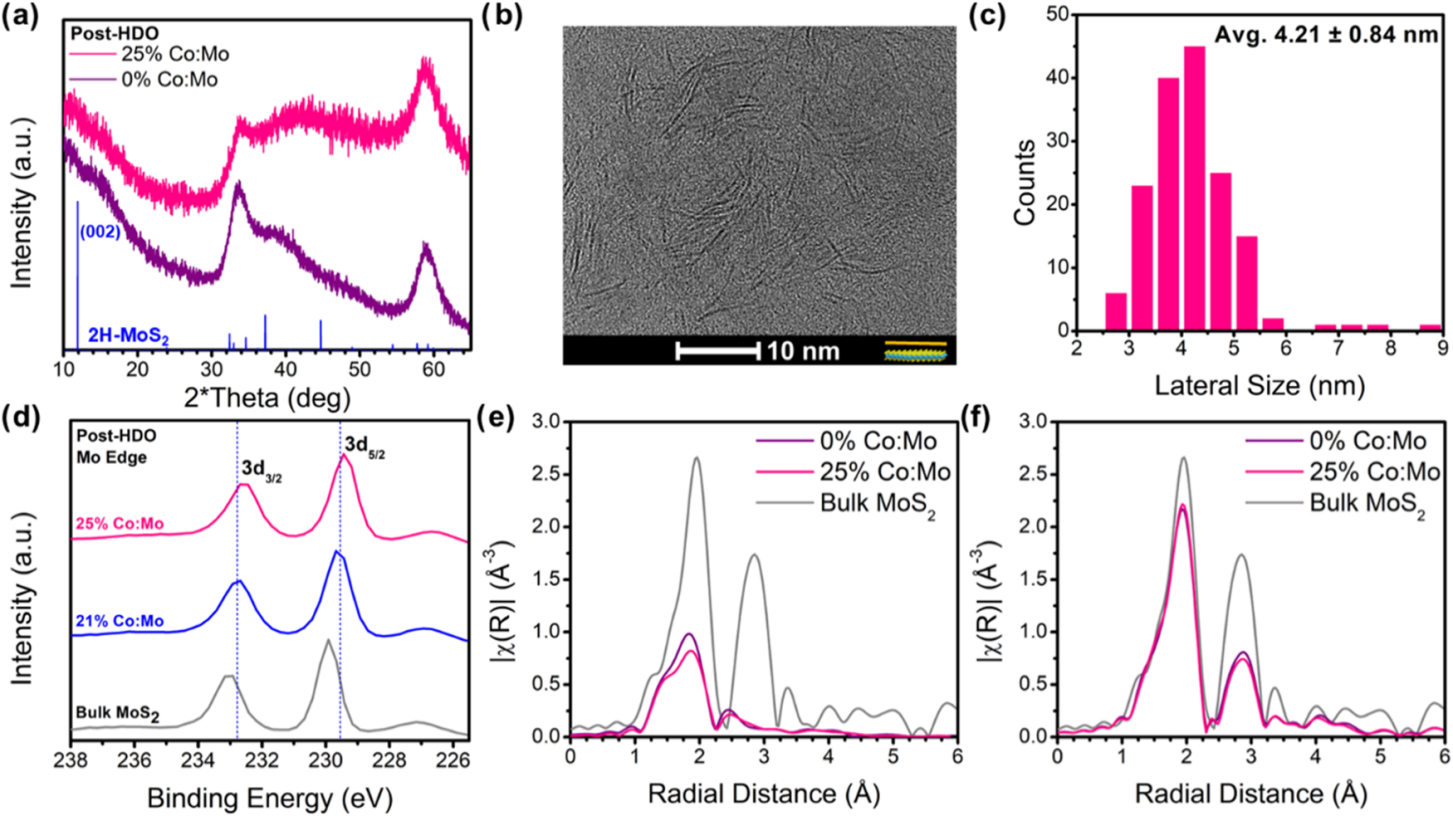
(a) X-ray diffraction patterns of cobalt-free MoS_2_ and 25% Co:Mo catalysts postuse in HDO, compared with a reference pattern for hexagonal MoS_2_,^43^ (b) high resolution transmission electron microscopy of 25% Co:Mo after use in HDO, with a model nanosheet shown edge-anchored to the surface revealing the side-on orientation and with an orange bar showing where lateral size is measured, (c) size distribution of nanosheets measured in 2b (sample size = 160 nanosheets), (d) X-ray photoelectron spectroscopy of the Mo edge of 21% and 25% Co:Mo after use in HDO. X-ray absorption spectroscopy data at the Mo K-edge for fresh (e) and post-HDO (f) catalysts.

X-ray photoelectron spectroscopy (XPS, Figure 2d) of post-HDO catalysts show the Mo electronic state with respect to Co concentration. We note a redshift in the Mo-edge 3d_5/2_ and 3d_3/2_ peaks compared to bulk MoS_2_, and further redshifting as Co loading increases, indicating a reduction in the Mo charge state by the addition of Co. A similar redshift is seen in the S-edge XPS spectra in both the 2p_1/2_ and 2p_3/2_ peaks compared with bulk MoS_2_ (Figure S9, Supporting Information). We further confirm the Mo–S structure by X-ray absorption spectroscopy (XAS) at the Mo K-edge. The data in Figure 2e,f show the extended X-ray absorption fine structure (EXAFS) in *r-*space before and after HDO, respectively. As we previously reported,^27^ we observe a mismatch in the EXAFS data between synthesized (fresh) nanosheets and bulk MoS_2_. While the first shell peaks (corresponding to Mo–S shell) are located where expected, the second shell is much shorter with significantly reduced magnitude and is attributed to Mo–Mo shell.^27,37^ This change is caused by the distortion of Mo atoms out of plane to accommodate the metastability of the 1T phase, leading to the distorted 1T’ phase.^36,38–40^ Post-HDO measurements agreed with bulk MoS_2_, indicating the structural change has occurred. By employing multiple structure probing techniques, we have confirmed that the same phase change from 1T’- to 2H-MoS_2_ occurs in HDO as it does in HDS.^27^ This change occurs rapidly, as just heating the catalyst up to reaction temperature shows a structural change that is similar to the post-HDO catalyst when probed in Mo K-edge (Figure S10, Supporting Information). The improved catalytic activity must originate from the nature of the Co atoms themselves; the 1T phase, which is more catalytically active, is not retained.^35^

One concern with TMD catalysts, particularly MoS_2_, is their stability under strong hydrogenation conditions. In HDS, a commonly reported reaction mechanism is the formation of vacancy sites on MoS_2_ *in situ*, as H_2_ scavenges S atoms from the catalyst surface to form H_2_S.^41,42^ These vacancies then accept S heteroatoms from the target organosulfur compound (i.e., thiophene), replenishing the catalyst. In the case of other hydrogenation reactions like HDO, where excess sulfur is not present, the concern lies that MoS_2_ may be completely or near completely stripped of its surface S atoms, depriving it of its catalytic activity over time. Figure 2f does not show any appreciable loss of average Mo–S coordination after 2 h of HDO, nor do we observe any growth in oxidation of the Mo atoms. The Mo–S shell does not change significantly during the reaction, either (see Figure S11 in the SI), suggesting that the catalyst structure is stable under HDO conditions. Figures S12 through S19 in the SI show theoretical fits in *r-*space and *k*-space of the Mo K-edge for each catalyst post-HDO, and relevant fitting parameters are recorded in Table S1.

Like the Mo K-edge, we also analyze the impact of oxidation on the Co state among various locations. XAS was performed on the Co K-edge of catalysts post-HDO to determine local structure around dopants, particularly after exposure to *p-*cresol at reaction conditions to see if the oxygen presence affected the structure. Figure 3a shows a slight shrinking in the first shell radial distance, which we attribute to a mix of Co–O and Co–S coordination, as Co concentration increases. By modeling these peaks in Artemis, as seen in Figure 3b, the Co–S paths still dominate compared to Co–O, indicating the Co is primarily coordinated with sulfur atoms on the MoS_2_ surface. As expected, Co bound to the edges (as observed at lower concentrations i.e., 10% Co:Mo), shows a higher coordination (3.7) than Co in catalysts with both edge and basal doping (25% Co:Mo), which has a Co–S coordination of 2.9. Conversely, the Co–O coordination increases slightly with Co concentration. This observation is likely due to the fact that oxygen may readily interact with the more active basal species, partially oxidizing these active centers. Bond lengths also followed the expected trend, where the introduction of basal Co reduced the average bond length as was calculated in a previous report.^27^ XANES (Figures S20 and S21) and *k-*space (Figure S22) of the Co K-edge compared with a cobalt­(II) oxide reference, as well as *R-*space (Figures S23 and S24) and *k*-space fits (Figures S25, S26, S27) of the Co–O and Co–S paths fitted in Artemis can be found in the SI. A table of relevant fitting parameters of the Co K-edge (Table S2) can be found in the SI, showing good fit to the local structure for each catalyst (*R*-factor less than 2%). Further understanding the behavior of Co, as well as the origin of this oxidation change, is supported by computation to fully grasp how oxygen interacts with the catalyst. The sharp peaks that arise at ∼779.4 eV in the post-HDO Co edge XPS (Figure 3c) are generally indicative of a CoMoS-type structure, in which isolated Co atoms are bound to S on the surface of MoS_2_.^44,45^ This is further supported by the lack of Co^0^ metal peaks (∼778 eV) and the formation of Co–S 2p_1/2_ peaks (∼795 eV), agreeing with the implications from EXAFS that the atoms are dispersed and primarily bound to sulfur.

**Figure 3:**
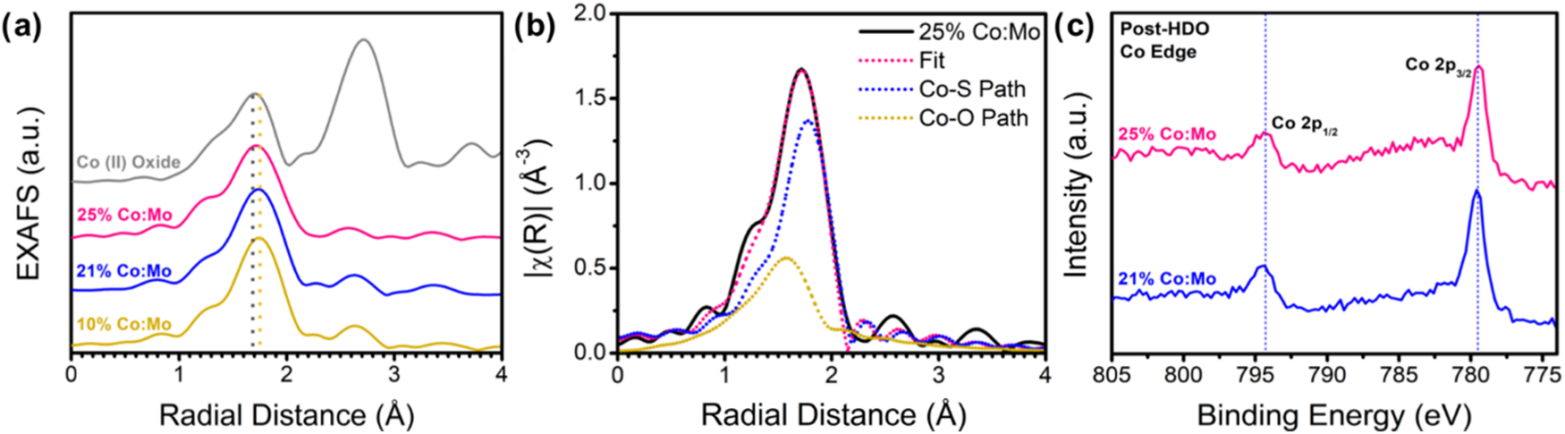
(a) Fourier transform magnitudes of *k* ^2^ -weighted EXAFS spectra at the Co K-edge of post-HDO catalysts compared with CoO reference, (b) first-shell fit of 25% Co:Mo catalyst EXAFS spectra at the Co K-edge for post-HDO, showing a mixed contribution of Co–S and Co–O bonding, (c) Co edge XPS of 21% and 25% Co:Mo catalyst post-HDO.

In a previous report,^27^ we performed DFT calculations to examine the preference of Co sites decorating different locations of the MoS_2_ nanosheet. It was determined that generally, the Co atoms prefer to dope the corner and edge sites of the nanosheet before doping the basal plane. Edge doping is nonideal for optimal catalytic activity, as it replaces active MoS_2_ edge sites with slightly more active Co sites but does not activate the inert basal plane. Beyond a dopant concentration determined by nanosheet size, the edges become saturated and basal plane doping begins. For these colloidal synthesized nanosheets, we determined saturation occurred before 21% Co:Mo was achieved.

### Computation of Catalyst Behavior

The interactive behavior of an aromatic alcohol with the MoS_2_ surface was examined by computing the energy of phenol as a model reactant adsorbed on pristine and Co-doped MoS_2_. We chose phenol for computational simplicity, as we do not expect significant impacts nor steric hindrance from the *p-*methyl substitution, focusing solely on reactant/catalyst interaction. Ideally, a selective catalyst should bind primarily at oxygen atom; direct adsorption of the aromatic ring can more easily lead to hydrogenation of the aromatic carbons, which is undesirable in HDO. Figure 4 depicts the various structures of phenol-MoS_2_ interaction and the relevant energies, including pristine MoS_2_ and with Co dopants in various locations. The unpromoted MoS_2_ yields a very slight preference for adsorption atop the basal plane, where van der Waals interactions occur between the basal plane and aromatic ring, (−0.50 eV) rather than the more active edge sites, where OH adsorption occurs (−0.46 eV, Figure S28). No products of ring hydrogenation (4-methylcyclohexanol, methylcyclohexane, etc.) were observed when using Co-free MoS_2_ nanosheets, thus we conclude that that such an interaction on the basal plane cannot activate the aromatic ring for hydrogenation or conversely, cannot activate hydrogen to hydrogenate the ring. Attachment of the OH group along the edges of the nanosheets likely drives the reaction in this case. By comparison, phenol binds more strongly to Co-promoted MoS_2_ at all sites compared with bare MoS_2_. Binding of the oxygen atom is favored, which supports the high selectivity toward preserving the aromatic structure of the final product. Co situated on the basal plane demonstrates a stronger binding energy than other locations. This supports the findings of Figure 1, where improvement by Co addition is nonlinear, and thus not all Co sites promote the reaction equally. Also given that we do not observe any significant formation of sulfur vacancies or undercoordination of Mo in the Mo K-edge EXAFS (Figures 2f and S12), we do not expect alternative, acid sites that may compete with the Co. We therefore conclude that the HDO is driven through a direct deoxygenation pathway over the Co site. The significant increase in HDO activity implies that it is not just an increase in the number of sites, but an increase in the relative activity of individual sites.

**Figure 4:**
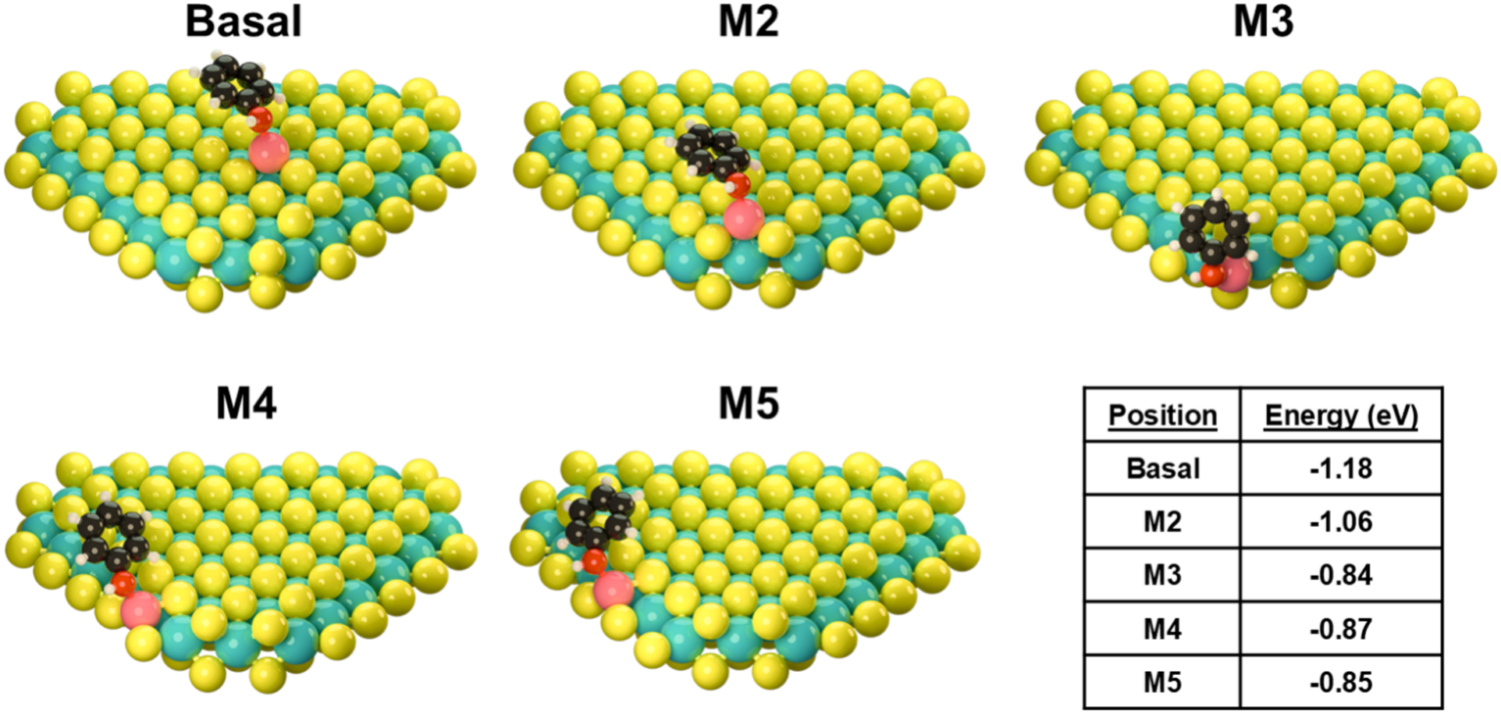
Examined locations of phenol adsorption on model Co-doped MoS2 nanosheets and their associated relative energies. Carbon is shown in black, hydrogen in white, and oxygen in red.

To understand the effects of product inhibition on Co activity, we computed the binding energy of oxygenated species to Co active centers, using H_2_O as the model oxygen product to (see structures in Figure S29 in SI). This result is compared with our previous computation using H_2_S adsorption as an analogue for HDS.^27^ Figure 5 depicts example structures of MoS_2_ decorated with single atoms of Co and their computed reaction product binding energies. As expected, the less favorable basal Co position interacts more readily with its environment, and thus it adsorbs both H_2_O and H_2_S more favorably than edge Co. This agrees with our observations that basal Co atoms are generally more active, per unit Co, than edge Co atoms. Interestingly, the stronger binding of H_2_S agrees with the increased Co–S coordination seen in the post-HDS EXAFS compared with the post-HDO catalyst (Tables S3 and S4, respectively). Conversely, the small amount of extra oxidation for basal Co indicates the H_2_O product desorbs readily and we are not excessively oxidizing the surface. In the case of HDS, basal Co was largely sulfided in excess, which dampened activity at higher Co concentrations. This does not hinder the HDO reaction, and thus 25% Co:Mo shows much greater activity as it activates the basal plane and is not poisoned by the products. Figure 5 summarizes the DFT results and models examples of structures for Co location as well as how H_2_O adsorbs to the Co surface.

**Figure 5:**
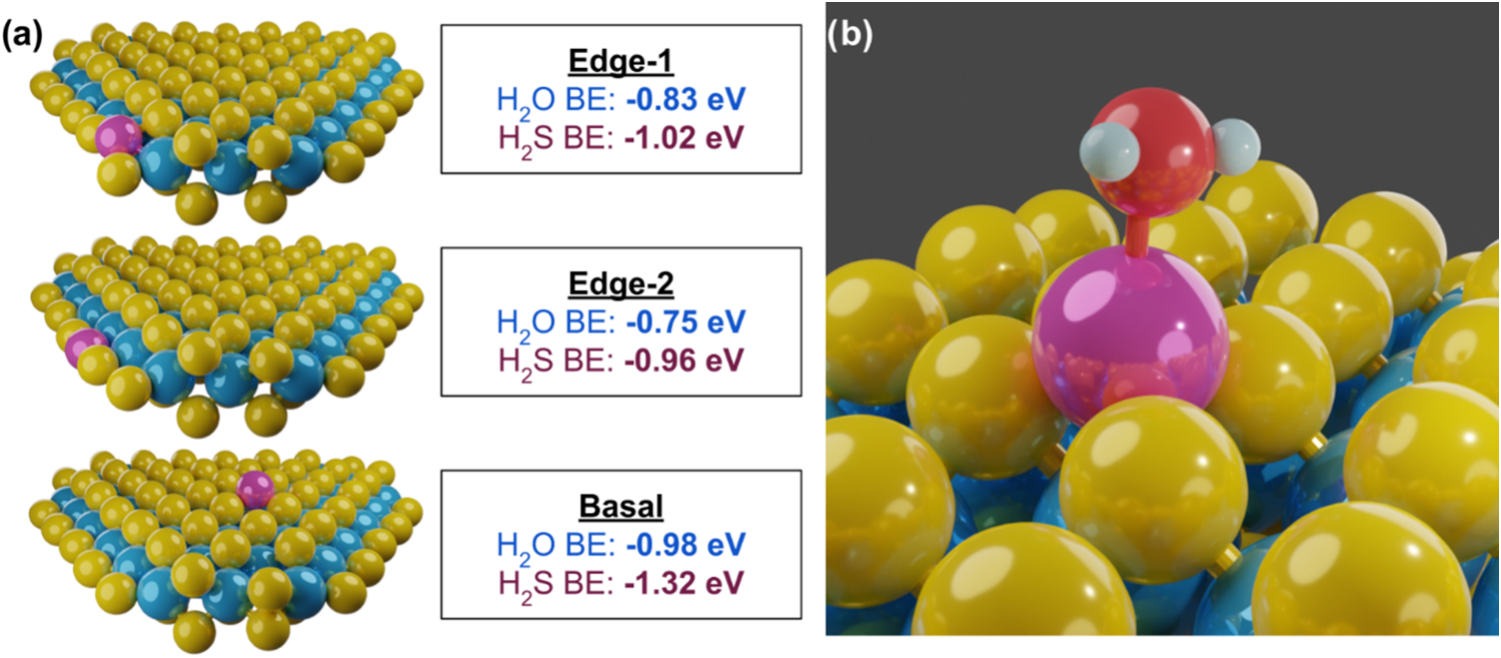
Examples of most expected Co locations as computed by DFT (a) and their associated binding energies of H_2_O and H_2_S.^22^ A closeup (b) of basal Co is shown in its preferred adsorption configuration of H_2_O. Hydrogen is shown in white, oxygen is shown in red.

Generally, a more negative binding energy favors adsorption, but it can also make desorption difficult and can even permanently bind the target molecule to the catalyst site.^46^ This can limit the effectiveness of sites or even deactivate sites if the new structure is less active or entirely inert. In this context, we have previously reported^27^ that H_2_S binding to basal Co was strong enough (compared to edge Co) to sulfidize the basal Co atoms, creating Co sites with one extra sulfur atom on average and limiting the kinetics of the reaction as more basal Co sites were created, observed via XAS and DFT. Desorption of H_2_O product, which has a lower binding energy, is less hindered, which promotes completion of the reaction and does not alter the structure of the Co atoms. Oversulfidation of the Co may also change its catalytic behavior, but this is not present in HDO. Excess oxidation is also not observed, indicating the stability of the dispersed single Co atoms anchored to the basal plane. In both cases, binding energy was stronger on basal Co than on edge Co. This may create the balance of adsorption strength such that adsorption on the edges is too weak to drive the reaction, but basal Co adsorbs and desorbs oxygen with the right balance to maintain the reaction kinetics.

Comparing the activities of lower and higher Co concentrations, we see that the expected saturation point (∼15–20% Co:Mo) appears in the resulting *p-*cresol conversion trends. This range, computed from the average nanosheet size in Figure 2b on the basis of available edge area and the preference of Co to bind to the edges before the basal plane (see Figure S30 and Table S5, Supporting Information), allows us to observe the difference between edge and basal Co sites. Loadings near this range (10%, 21% Co:Mo) have predominantly edge Co, with some basal Co present on 21% Co:Mo per XAS analysis (Figure S24), while higher loadings (25%, 39% Co:Mo) exhibit more basal Co. Thus, the large gap in activity between 21% and 25% Co:Mo likely results from the efficiency of basal Co in creating new active sites on the nanosheet, rather than replacing active edge sites. The range of approximately 15–20% arises from the size distribution of the nanosheets, and it is probable that some basal Co also exists within the population of 21% Co:Mo catalysts. However, we presume it has a predominantly higher ratio of edge sites to basal sites compared with 25% Co:Mo, explaining the lower per site efficiency as seen in Figure 1d,e. In HDS, 21% and 25% Co:Mo had shown similar activity, by contrast to what is observed in HDO. This is a result of the faster desorption of H_2_O from the basal Co atoms present beyond 21% Co:Mo, relative to H_2_S, which implies that there is less kinetic limitation and therefore more turnovers per unit basal Co. This provides a better realization of the role that basal Co plays in hydrogenolysis reactions, particularly since ligand-based crowding of Co at excessive loadings (39% Co:Mo) show far lower activities. This is likely due to steric hindrance by surface ligand and Co complex species. Combining DFT with XAS and reactor studies allows us to better comprehend the role that basal Co plays in this reaction, particularly the effectiveness of ligand-directed Co single atoms on the surface of MoS_2_.

The single Co atoms remain isolated, as no cobalt oxide clusters were observed. We therefore treat dispersed atoms of Co as the catalyst center in this system as the center of interest. H_2_S desorption is slow compared with H_2_O due to its more negative binding energy, which leads to sulfur remaining on Co after thiophene has been decomposed. The less negative binding energy of H_2_O leads to both a conversion of *p-*cresol and a relatively complete desorption of the products, improving the reaction rate on basal Co. From these findings, it is apparent that while edge doping with Co can improve the activity of MoS_2_ for HDO, doping of the basal plane is key to optimizing its activity.

## Conclusion

The preceding sections demonstrate the successful colloidal synthesis of Co-MoS_2_ nanosheets variably decorated *in situ* with single Co atoms and the subsequent study of the relationship between local Co structure and HDO activity. Using spectroscopic techniques, we have determined that nanosheet size and Co concentration each play a crucial role in directing the location of Co and its subsequent catalytic behavior. Co prefers to adsorb along the nanosheet edges; once a saturation point is reached (assisted by directing oleic ligands), adsorption on the basal plane becomes favorable until a crowding point is reached, yielding optimal activity around 25% Co:Mo. Thus, to activate the basal plane of MoS_2_ and create more active Co species, the threshold of edge-doping must be overcome. We also observe that the edge saturation point of MoS_2_ is less than the available number of edge sites, possibly dictated by steric and/or electrostatic hindrances. While Co-doped MoS_2_ edges are more catalytically active than undoped edges, the Co atoms on the basal plane are demonstrably more active than edge-doped Co. The presence of this optimum and the details of these findings provide insight into how doped TMDs behave in hydrogenolysis and can inform the design of next generation catalyst materials tailored to respective applications. While these results are specific to Co-doped MoS_2_, the analyses we performed are a guideline that can be extended to other 2D TMD materials and dopants to help realize new phenomena in catalytic activity.

## Experimental Section

### Colloidal Synthesis of Co-MoS_2_ Nanosheets

Co-MoS_2_ nanosheets were prepared via a solvothermal hot-injection method, using a method that we have previously published.^27^ Nanosheets were synthesized by heating MoCl_5_ in oleylamine on a Schlenk line, after degassing with standard Schlenk line techniques. This was followed by swift injection of bis­(trimethylsilyl)­sulfide (TMS_2_S) to nucleate MoS_2_, then by an injection 3 min later of Co-Oleate prepared *in situ*. The resulting nanosheets were washed three times with a mix of hexane, acetone, and methanol and then dried under vacuum overnight and stored in a nitrogen-filled glovebox.

### Hydrodeoxygenation of *p*-Cresol

HDO was performed using a 316 stainless steel stirred autoclave benchtop reactor from Parr Instruments (Model 4564, 160 mL chamber volume). The reaction solution was prepared containing 800 mg *p-*cresol, 800 mg decane (inert reference), and 80 mL of 1,2,3,4-tetrahydronaphthalene (tetralin). An aliquot of 0.5 mL of this solution was analyzed via gas chromatography–mass spectrometry using a Shimadzu QP2010 GC-MS. The sample was analyzed three times to determine the average concentration of *p-*cresol.

The reaction solution was then sonicated for 10 min following the addition of 80 mg of catalyst, then transferred to the reactor. The reactor chamber was sealed and degassed by filling with 95%N_2_/5%H_2_ gas up to 13.8 bar_g_, then vented and repeated twice more. The chamber was then pressurized with 95%N_2_/5%H_2_ gas to 17.2 bar_g_ and heated to 300 °C with a mixing speed of 300 rpm. Using a sample collection vessel (Parr Instruments, Model 4351), 0.5 mL aliquots were removed from the reactor periodically, up to 120 min of runtime. The sample vessel was quickly cooled by passing water through a cooling jacket. The aliquots were filtered to remove the catalyst, and the filtrate was then analyzed by GC-MS following the same procedure as the prereaction mixture sample.

### Computational Methods

All calculations (unless specified otherwise) were carried out for a single-layer freestanding, unsupported hexagonal nanoparticle model of MoS_2_ with Mo-edge containing six Mo atoms (including the corner atoms) while the S-edge contains three Mo atoms (including the corner atoms), reflecting the typical truncated triangular shape of a single-layer particle observed in published scanning tunneling microscopy (STM) experiments.^13^ Two layers of S atoms sandwich the Mo layer such that they are in the trigonal prismatic positions characteristic of the 2H phase of MoS_2_. All figures in this paper with MoS_2_ structures and adsorption configurations show Mo atoms in blue, S atoms in yellow, Co atoms in pink, O atoms in red, and H atoms in white. We further assumed that the sulfur edge is 100% S-decorated while the metal edge is 50% S-decorated, consistent with ab initio phase diagrams and STM observations.^47^ A single Co atom was included in the calculations in many cases (in different locations) to represent cobalt decoration of MoS_2_ basal plane. While the exact decoration of metal and sulfur edges of MoS_2_ nanoparticles are dependent on the reaction conditions and may include oxygen atoms (in the presence of water)^48^ and the freestanding layer does not capture stacking of MoS_2_ and support interactions, we posit that the given model is adequate to provide a comparative analysis of different plausible sites along the periphery versus the basal plane for adsorption and reaction.

The calculations were carried out using VASP,^49,50^ a plane wave periodic DFT code. Generalized gradient approximation and projected augmented wave (PAW) potentials^51^ were used with PBE exchange correlation functional^52^ and the D3 Grimme dispersion correction.^53^ All calculations were carried out in a box that had at least 10 Å of vacuum between two images in all directions. Spin polarization is included in all calculations: the effect of spin was found to be negligible (∼0.01 eV on the vacancy formation energy). Plane wave and density wave cutoffs of 400 and 645 eV were used, respectively. A Gaussian smearing of 0.05 eV was used, and the energies were extrapolated to 0 K. Only γ-point sampling was used in view of the large dimensions of the supercell. The convergence criterion for geometric relaxation was set to 0.02 eV/A. The energies computed using VASP are not reported as such; only energies relative to a reference are presented. The binding energy of phenol and water (H_2_O) adsorption, BE_H_2_O/Phenol_, was computed using the following equation

1 $$BE_{H_{2}O/\mathrm{Phenol}}=E_{H_{2}O/\mathrm{Phenol}}-E_{\mathrm{NS}}-E_{H_{2}O\left(g\right)/\mathrm{Phenol}\left(g\right)}$$

where *E*_H_2_O/Phenol_ is the energy of H_2_O or phenol adsorbed onto the nanosheet, *E*_NS_ is the energy of the free nanosheet, and *E*_H_2_O(g)/Phenol(g)_ is the energy of H_2_O/Phenol in the gas phase.

## Supplementary Material



## Notes

Hydrodeoxygenation reaction pathways; supplemental reactor and catalyst recycle testing data; turnover frequency computation; supplemental X-ray diffraction data; supplemental high resolution transmission electron microscopy data and population counts; supplemental X-ray photoelectron spectroscopy; supplemental X-ray absorption spectroscopy data and EXAFS peak fitting; details on computational models and site count estimations (PDF)
