# Supplementary material for: Basal Plane Doping to Activate Colloidal MoS2 Nanosheets for Catalytic Hydrodeoxygenation of para-Cresol
Source: ACS Appl Mater Interfaces. 2026 Apr 4;18(14):20838–47. doi: 10.1021/acsami.5c24853 (PMC13088027; doi:10.1021/acsami.5c24853)
Supplement: Supplementary file 1 [file am5c24853_si_001.pdf]

Supporting Information

Basal Plane Doping to Activate Colloidal MoS_2 Nanosheets for Catalytic Hydrodeoxygenation of *para*-Cresol

Steven L. Farrell[1], Noah Osinski[1], Ingrid J. Paredes[1], Amani M. Ebrahim[2], Hao Xian Zheng[1], Yuchen Zhang[1], Christopher Oyuela[1], Lu Ma[4], Steven N. Ehrlich[4], Srinivas Rangarajan[3]*, Anatoly I. Frenkel[2,5]*, and Ayaskanta Sahu[1]**

[1]Department of Chemical and Biomolecular Engineering, New York University, Brooklyn, New York 11201, USA.

[2]Department of Materials Science and Chemical Engineering, Stony Brook University, Stony Brook, New York 11794, USA.

[3]Department of Chemical and Biomolecular Engineering, Lehigh University, Bethlehem, PA 18015, USA.

[4]National Synchrotron Light Source II, Brookhaven National Laboratory, Upton, New York 11973, USA.

[5]Chemistry Division, Brookhaven National Laboratory, Upton, New York 11973, USA.

**Corresponding authors: sf2957@nyu.edu; srr516@lehigh.edu; anatoly.frenkel@stonybrook.edu; asahu@nyu.edu*

Supporting Information (SI)

Characterization Methods

X-ray Diffraction (XRD)

Samples were characterized with XRD using a Bruker AXS D8 Discover GADDS Microdiffractometer at New York University's Shared Instrument Facility. All samples were prepared by drop-casting from cyclohexane and drying on clean 1-cm^2 glass slides, and were measured with a Cu Kα source.

High Resolution Transmission Electron Microscopy (HRTEM)

HRTEM was performed using a 200 kV FEI Titan Themis Scanning TEM. Samples were prepared by dropcasting dilute samples of nanosheets in cyclohexane (fresh samples) or dimethylformamide (post-HDS) onto carbon coated copper grids and drying at 80 °C.

X-ray Photoelectron Spectroscopy (XPS)

XPS for examining Co, Mo, and S was performed using a Physical Electronics Versaprobe II XPS. Spectra were collected using an Al Kα source set to 49.4 W and 14.87 keV (250 meV resolution) with a 200 μm beam diameter. The survey pass energy was set to 117.40 eV, while the elemental pass energies were set to 29.35 eV. Samples were prepared by pressing powder pellets into double-sided tape on a silicon substrate. Prior to analysis, spectra were corrected by shifting the C_{1s} peak to 284.8 eV.

X-ray Absorption Spectroscopy (XAS)

Samples were measured at Brookhaven National Laboratory using the National Synchrotron Light Source-II (NSLS-II), Quick X-ray Absorption and Scattering (QAS, 7-BM) beamline. For hard X-ray energy measurements at QAS, samples were smeared onto clear adhesive tape, folded up to 8 times, and measured at the Co and Mo K-edges in fluorescence and transmission modes, respectively. The collected XAS data were then analyzed using the Demeter software package; the edge-step normalization to obtain XANES spectra, and background subtraction for EXAFS spectra, were performed in ATHENA, while modeling of the EXAFS data for Co and Mo K-edges was performed in ARTEMIS.[1] Parameters used in the fits include an amplitude factor of 0.76 (determined by fitting Co foil) and a *k*-weighting of 2. The R-range was 1.0-2.3 Å, and the *k*-range was 2.0-12.0 $Å^{-1}$ (dk = 2).

Gas Chromatography-Mass Spectroscopy (GC-MS)

Samples taken from the reaction mixture were analyzed using a GC-2030 gas chromatograph and GCMS-QP2020 NX gas chromatograph-mass spectrometer from Shimadzu. A 0.2-μL volume of analyte was injected at a column temperature of 40°C using a Shimadzu AOC-20i autosampler, then after one minute the column temperature increased to 250°C over 25 min. Prior to analysis, a calibration curve of the area ratio between thiophene and the n-decane reference was prepared using solutions of known concentrations. The concentrations of *p*-cresol in the mixture before and after reaction were measured using the area under the identified peaks in the chromatograph, and the percent conversion of *p*-cresol was calculated for each reaction run as follows:

$$X_{p-Cresol} = \frac{C_{p-Cresol,Before} - C_{p-Cresol,After}}{C_{p-Cresol,Before}}$$

Hydrodeoxygenation of p-Cresol

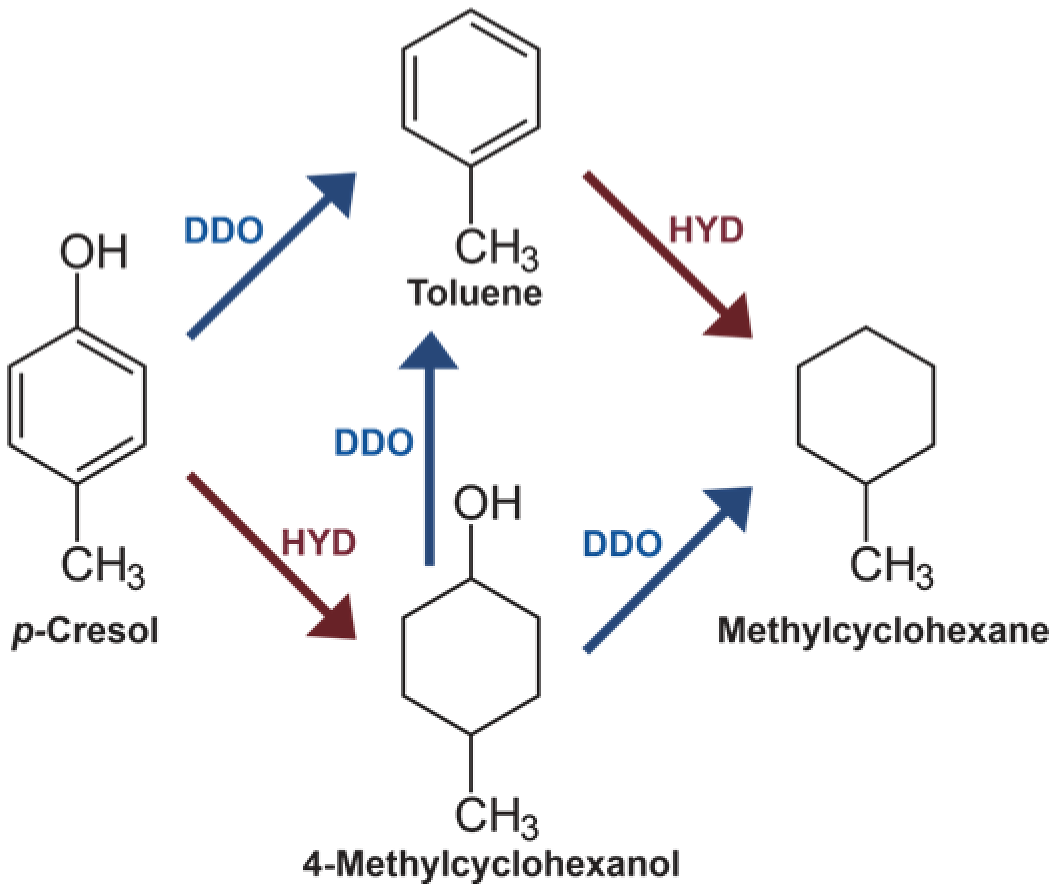

Figure S1. The expected reaction scheme for hydrodeoxygenation of *p*-Cresol, which can follow several pathways to create aromatic products, such as toluene, or less desired hydrogenated products, such as methylcyclohexane.

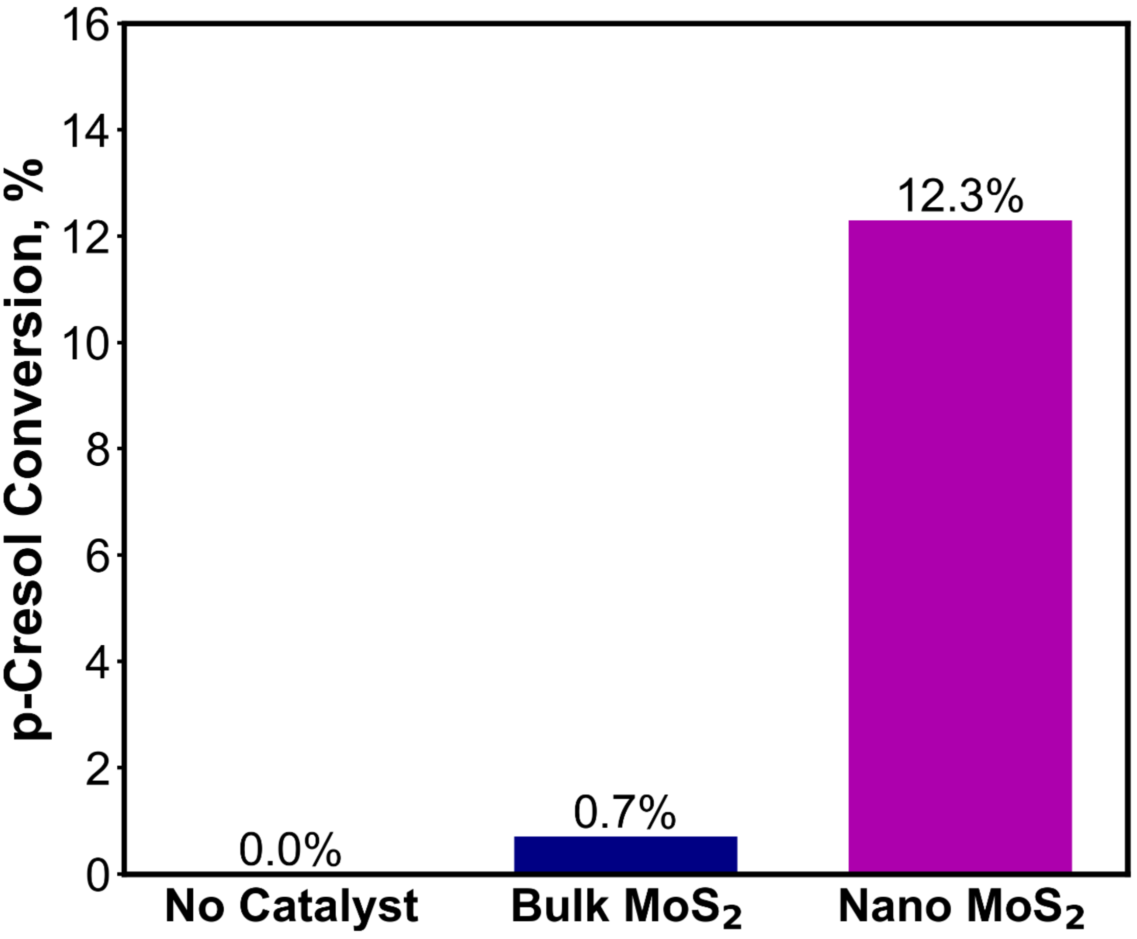

Figure S2. p-Cresol conversion activity without catalyst compared with that of bulk and nanoscale MoS_2 after a reaction time of 2 hrs (80 mL tetralin, 300°C, 250 psi). There is a sizable increase in activity moving to the nanoscale, as this generally creates sheets with more edge surface area.

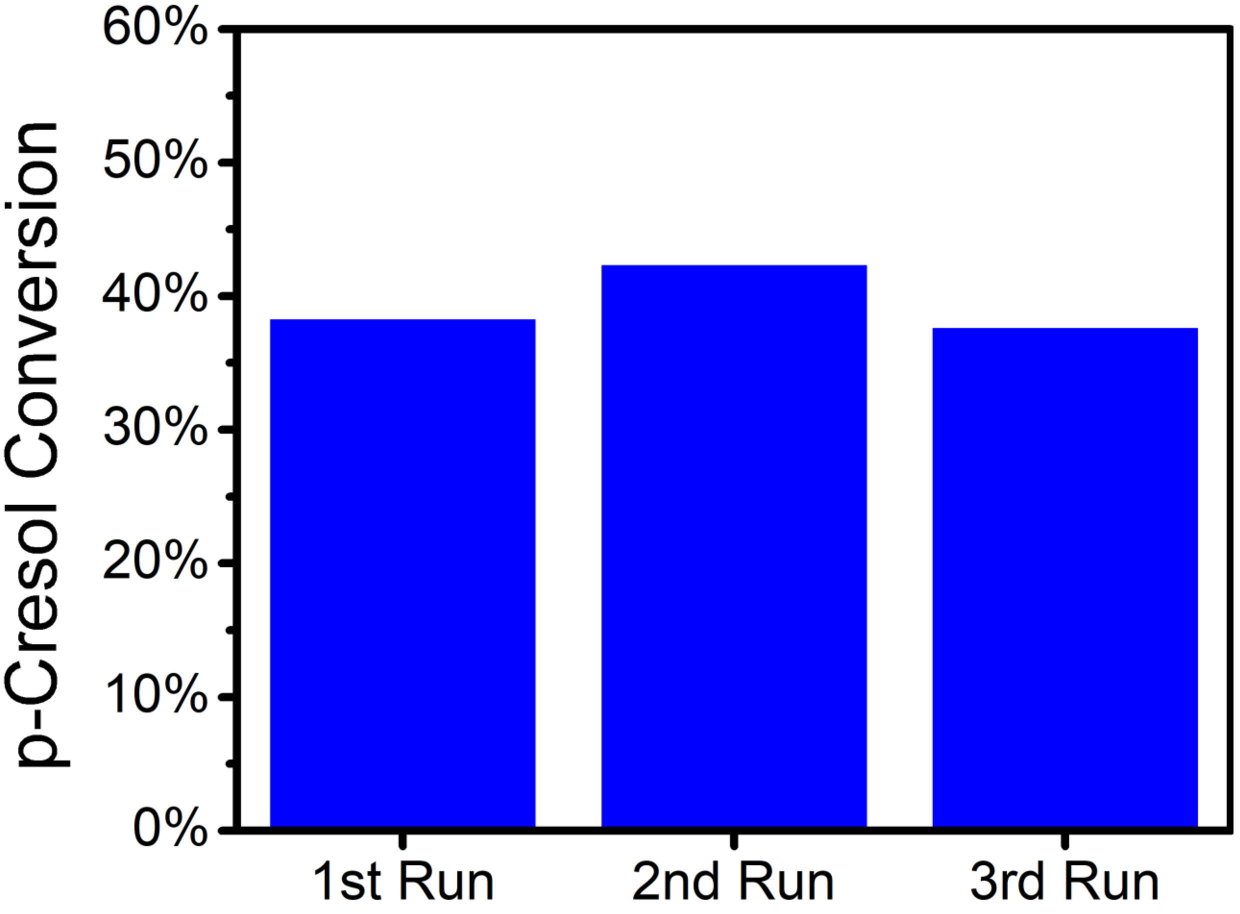

Figure S3. p-Cresol conversion activity of 25% Co:Mo MoS_2 catalyst after multiple reaction runs reusing the same catalyst. Little difference in the activity was observed. In these cases, 20 mg of catalyst was used with 500 mg of *p*-cresol, and no aliquots were taken over the 2hr reaction time.

Turnover Frequency Computation

Figure 1e in the main text uses the number of moles of potential sites as the basis for turnover frequency computation. Per our previous geometric structure computation (see ref. 26 in main text), we estimate an average of 36 edge site atoms for truncated triangular sheets. Combining this with our DFT and XAS observations of, and assuming decorated Co cannot sit next to another decorated Co (per XAS results and steric considerations), we compute that a Co:Mo ratio of 16.2% is the saturation point for the nanosheet edges. Assuming that Co covers an Mo site on the edge but creates a new site on the basal plane, we therefore can compute the number of sites per nanosheet, as used in Figure 1f, as follows:

If Co:Mo ≤ 16.2%: $N_{sites} = N_{edge\ Mo}$,

If Co:Mo > 16.2%: $N_{sites} = N_{edge\ Mo} + N_{total\ Mo} * (X_{Co:Mo} - 0.162)$

The quantity $N_{edge\ Mo}$ refers to the number of Mo atoms along a single nanosheet's perimeter. $N_{total\ Mo}$ refers to the total number of Mo atoms present in a single nanosheet. This is based on the assumption that a Co atom on the edge blocks one Mo atom, thus the number of edge sites does not change. However, any Co in excess of the saturation point (16.2%) would likely adsorb to the inert basal plane, meaning it creates an additional site.

The turnover frequency improvement per unit Co in Figure S1 below is calculated as follows:

$$TOF = \frac{N_{p-Cresol_{CoMoS_2}} - N_{p-Cresol_{MoS_2}}}{N_{Co} * t}$$

$N_{p-Cresol_{CoMoS_2}}$ is the number of moles of *p*-cresol converted using a Co-doped MoS_2 catalyst. $N_{p-Cresol_{MoS_2}}$ is the number of moles of *p*-cresol converted using nanoscale MoS_2 (0%

Co:Mo). N_{Co} is the number of moles of Co present in the Co-doped MoS_2 catalyst. t is the time of the reaction, in hours.

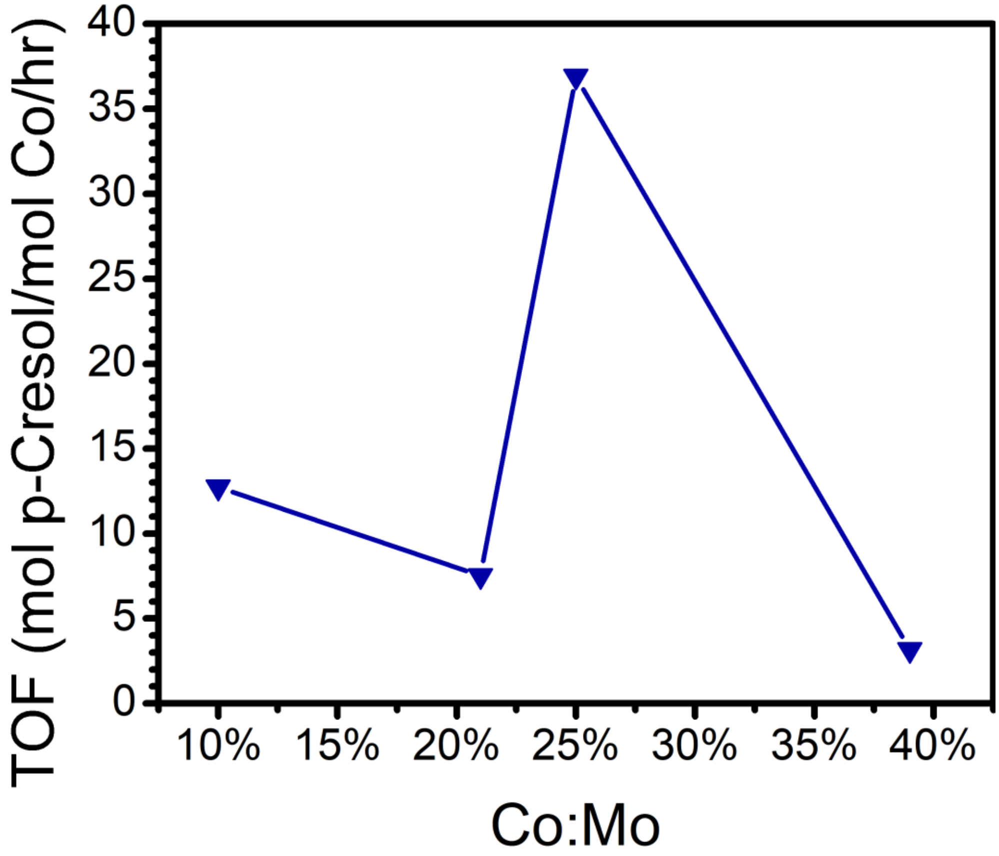

Figure S4. TOF improvement per mol Co basis, using the calculation procedure described above. The influence of MoS_2 edge sites is removed in order to emphasize the efficiency of each additional cobalt atom.

X-ray Diffraction

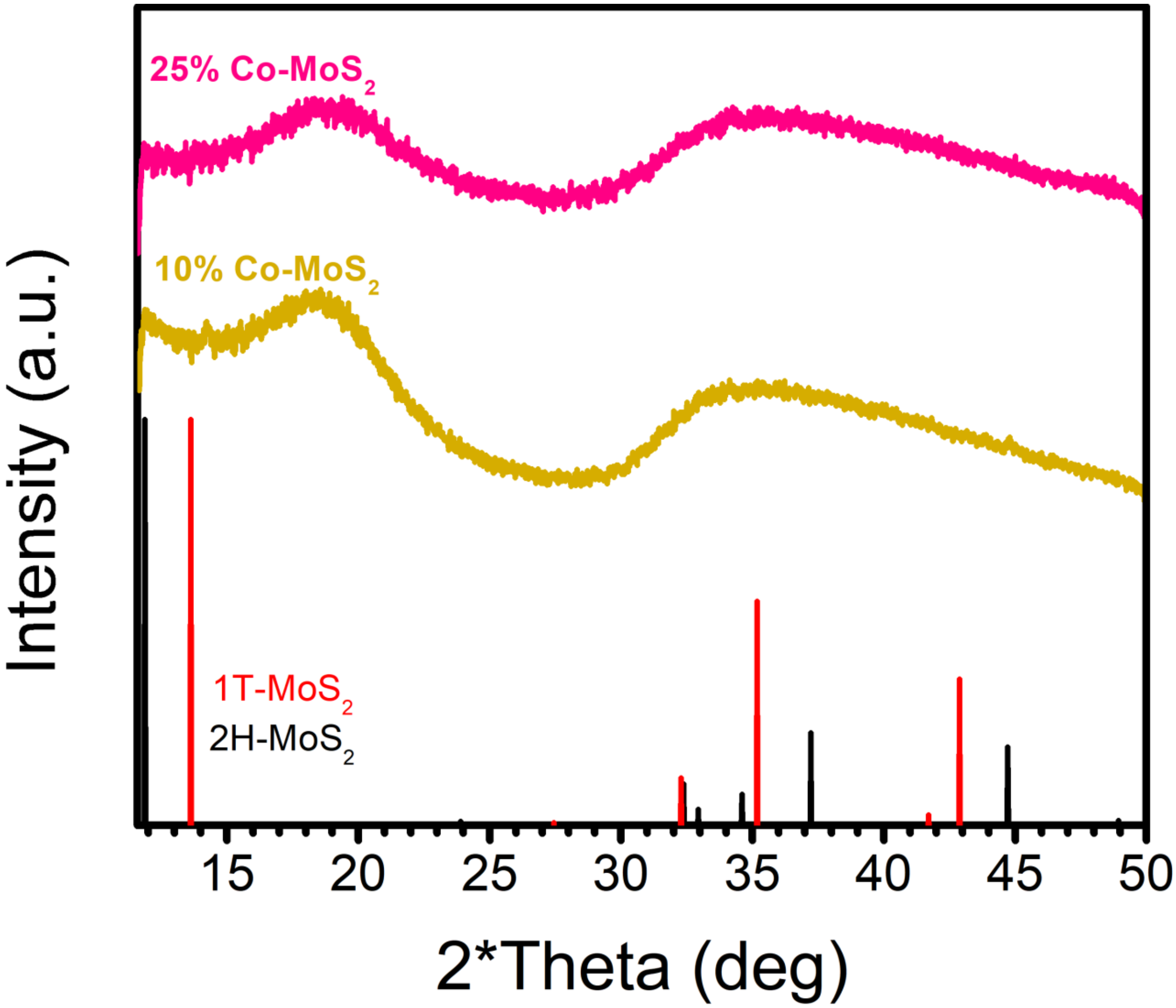

Figure S5. X-ray diffraction pattern of the as-synthesized fresh catalyst before HDO. Reference peaks are derived from CIF files computed from Materials Project.[2] 2H-MoS_2: MP-2815, 1T-MoS_2: MP-1238797.

High Resolution Transmission Electron Microscopy

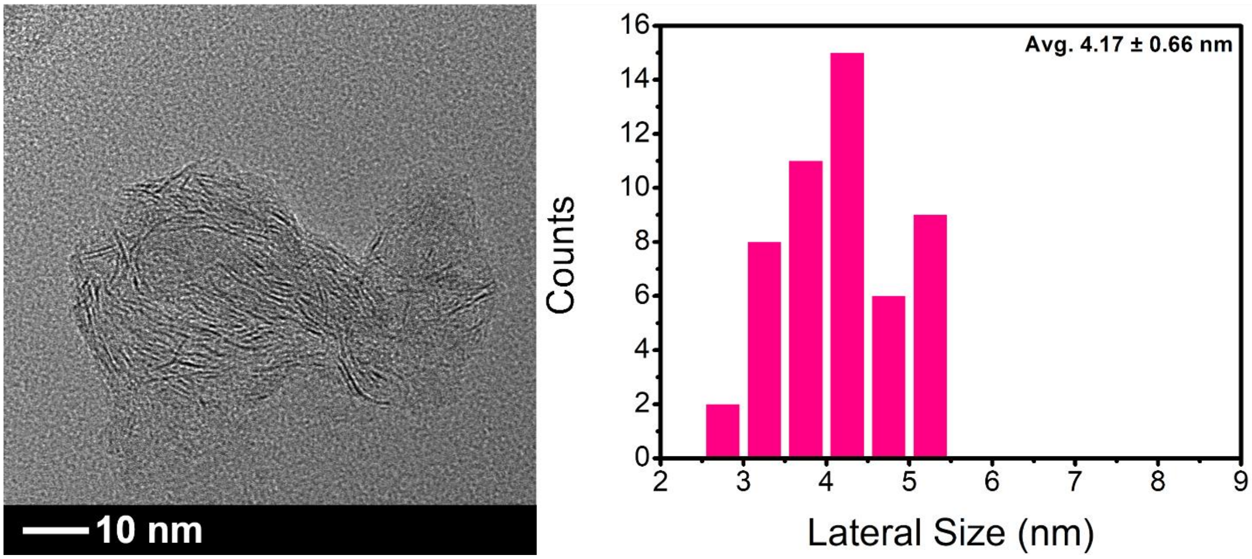

Figure S6. HRTEM of post-HDO 25% Co:Mo MoS_2 nanosheets. Nanosheets are seen in the side-on position (dark lines), of which the average length is measured. Population size = 51 nanosheets.

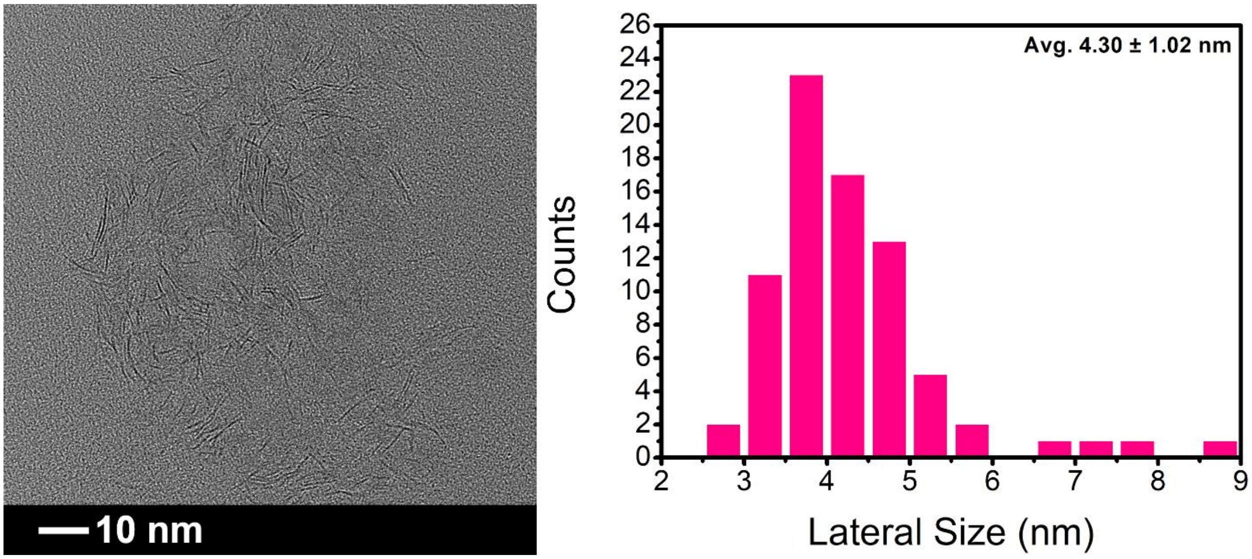

Figure S7. HRTEM of post-HDO 25% Co:Mo MoS_2 nanosheets. Population size = 77 nanosheets.

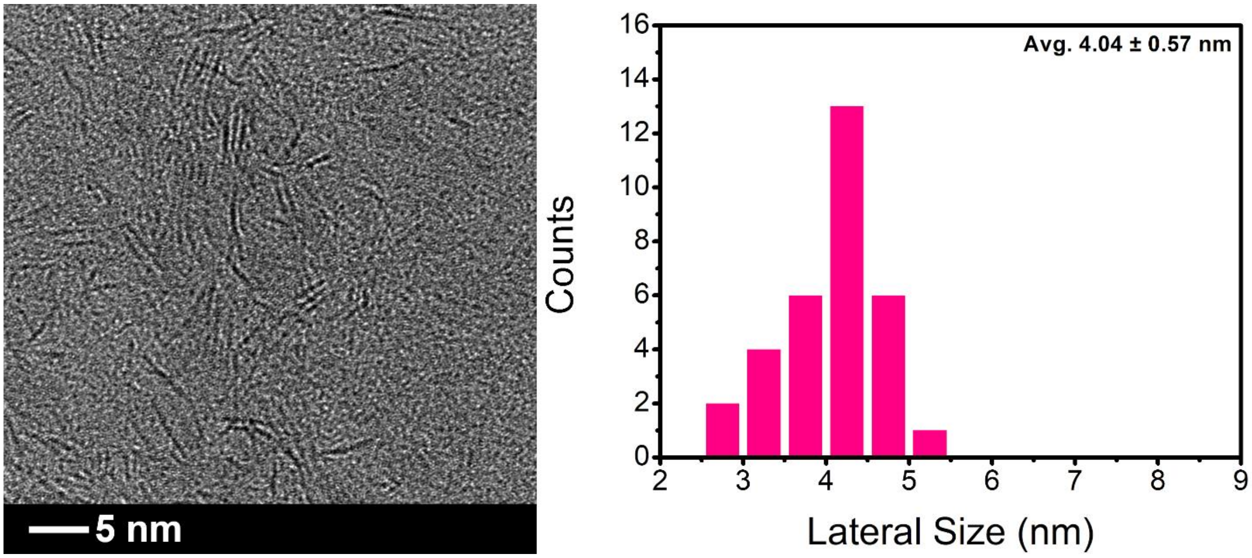

Figure S8. HRTEM of post-HDO 25% Co:Mo MoS_2 nanosheets. Population size = 32 nanosheets.

X-ray Photoelectron Spectroscopy

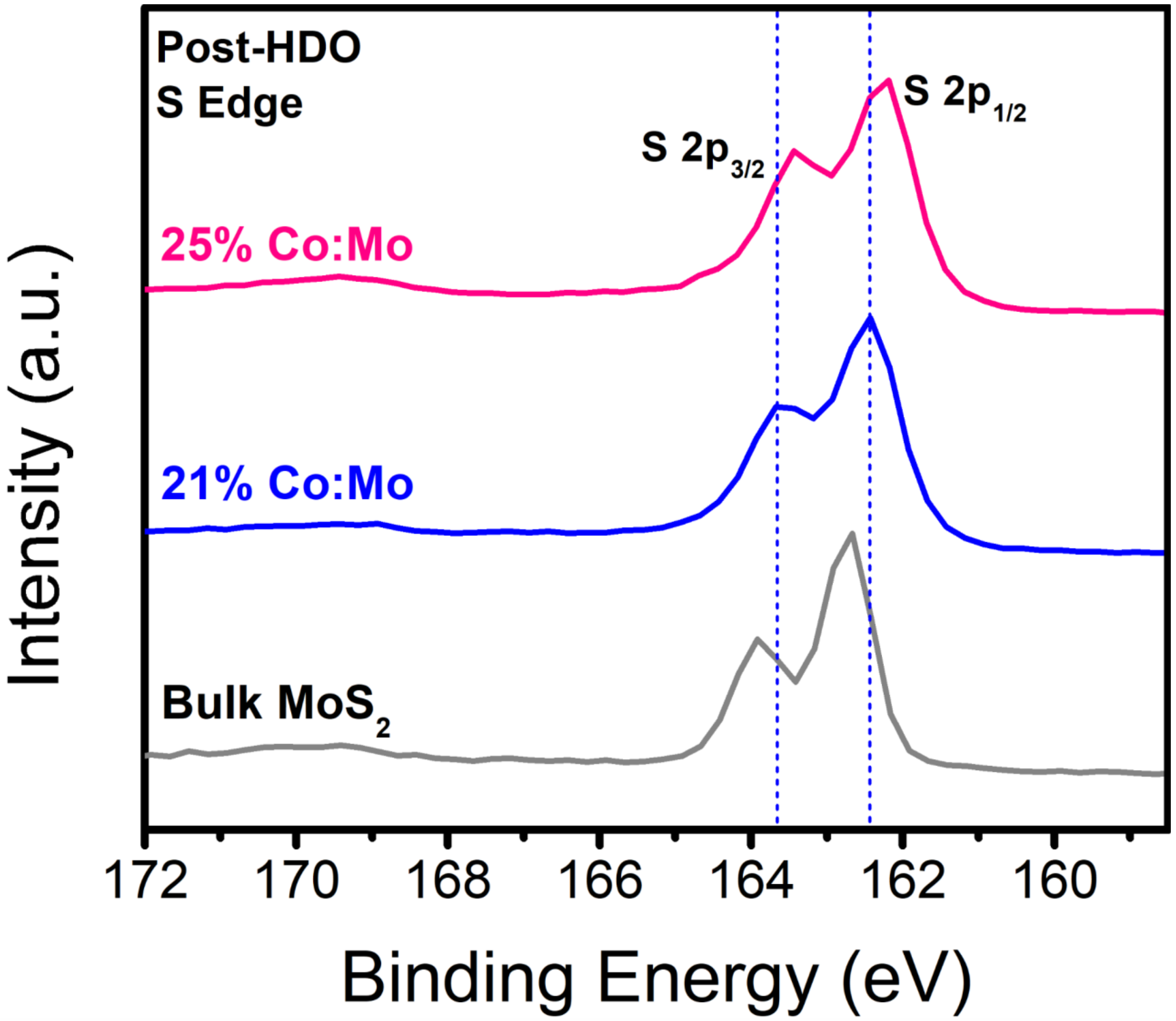

Figure S9. S edge X-ray photoelectron spectroscopy of bulk MoS_2 compared with 21% and 25% Co:Mo post-HDO catalysts. The addition of Co demonstrates a general redshift in the binding energy of the $2p_{1/2}$ and $2p_{3/2}$ peaks.

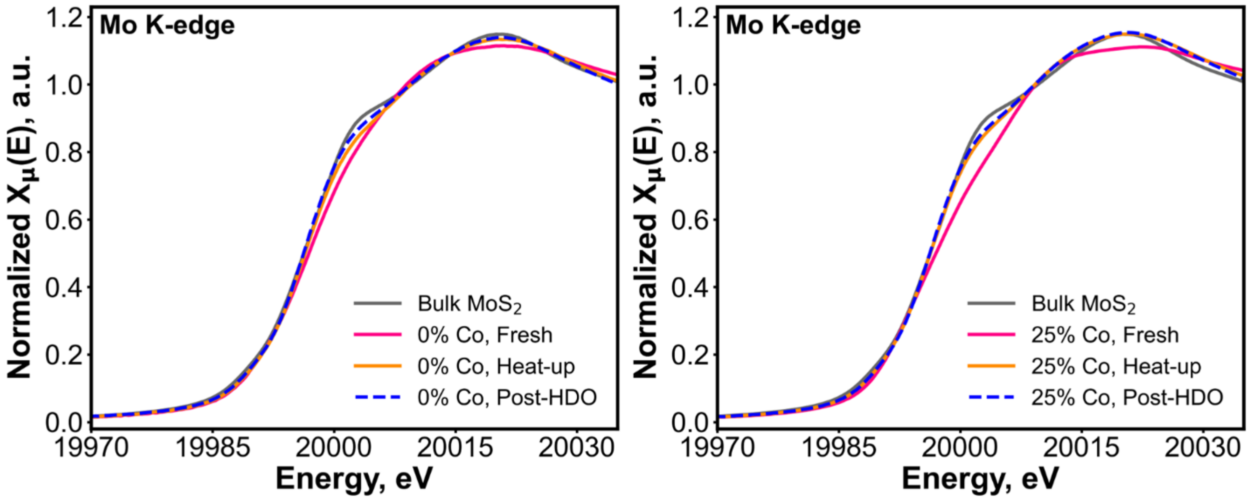

Figure S10. Mo K-edge XANES of 0% Co:Mo (left) and 25% Co:Mo (right) catalysts. For the "Heat-up" material, the reactor was run by heating to the reaction temperature (300°C) and then immediately cooled upon reaching the target temperature. For the "Post-HDO" catalyst, the reactor was run for a full 2 hours at the reaction temperature. We note that there is little difference between bulk MoS_2 and both catalysts after the heat-up step, so we conclude that the Mo state of the catalyst during the majority of the reaction (2 hours) are fairly similar regardless of Co loading.

X-ray Absorption Spectroscopy

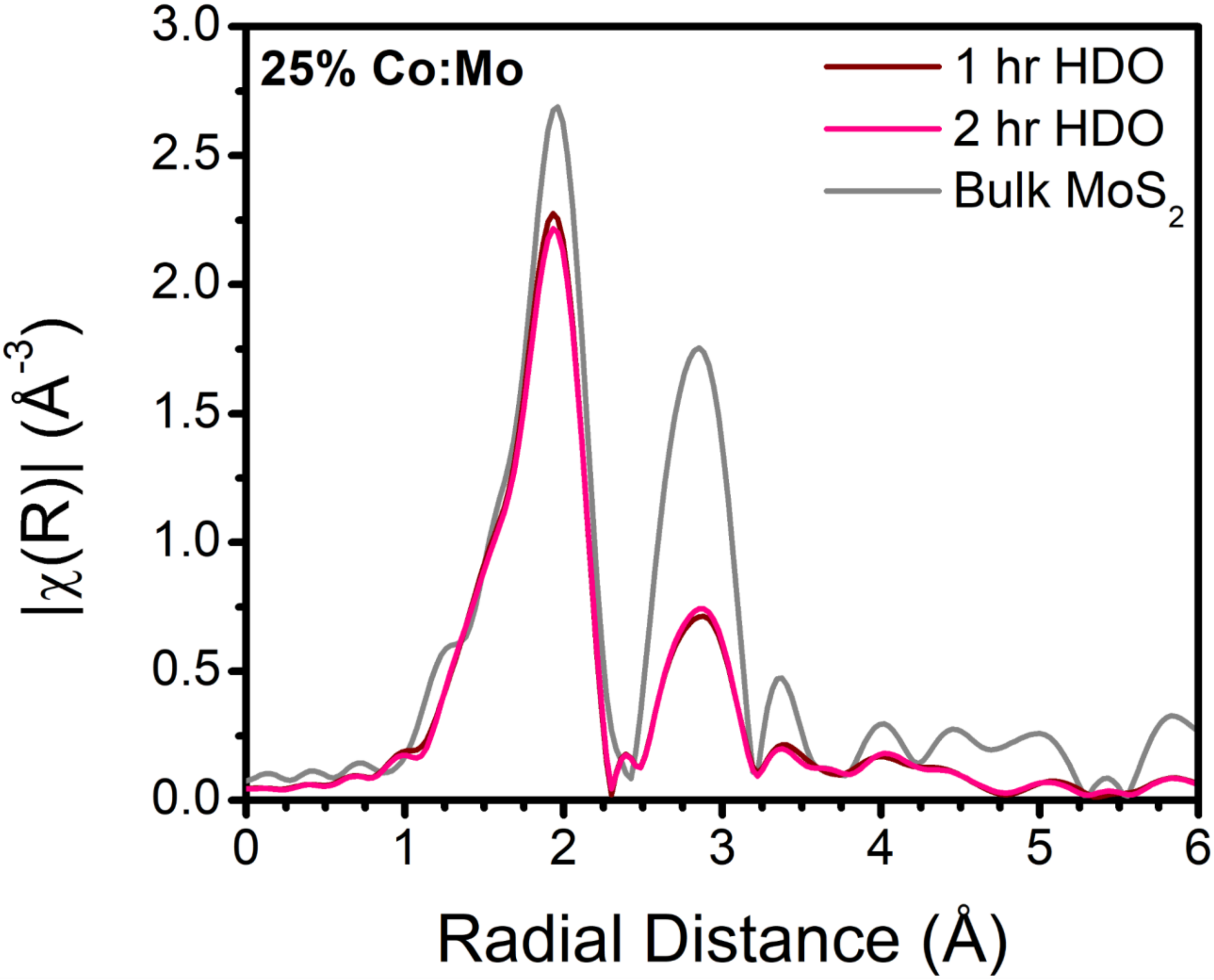

Figure S11. Fourier transform magnitudes of the k^2-weighted, *Ex situ* Mo K-edge EXAFS data of 25% Co:Mo samples fresh after 1 hr and 2 hrs under HDO conditions. The similar features, particularly in the first shell, indicate that the nanosheet sulfur atoms are not heavily scavenged by H_2 under reaction conditions.

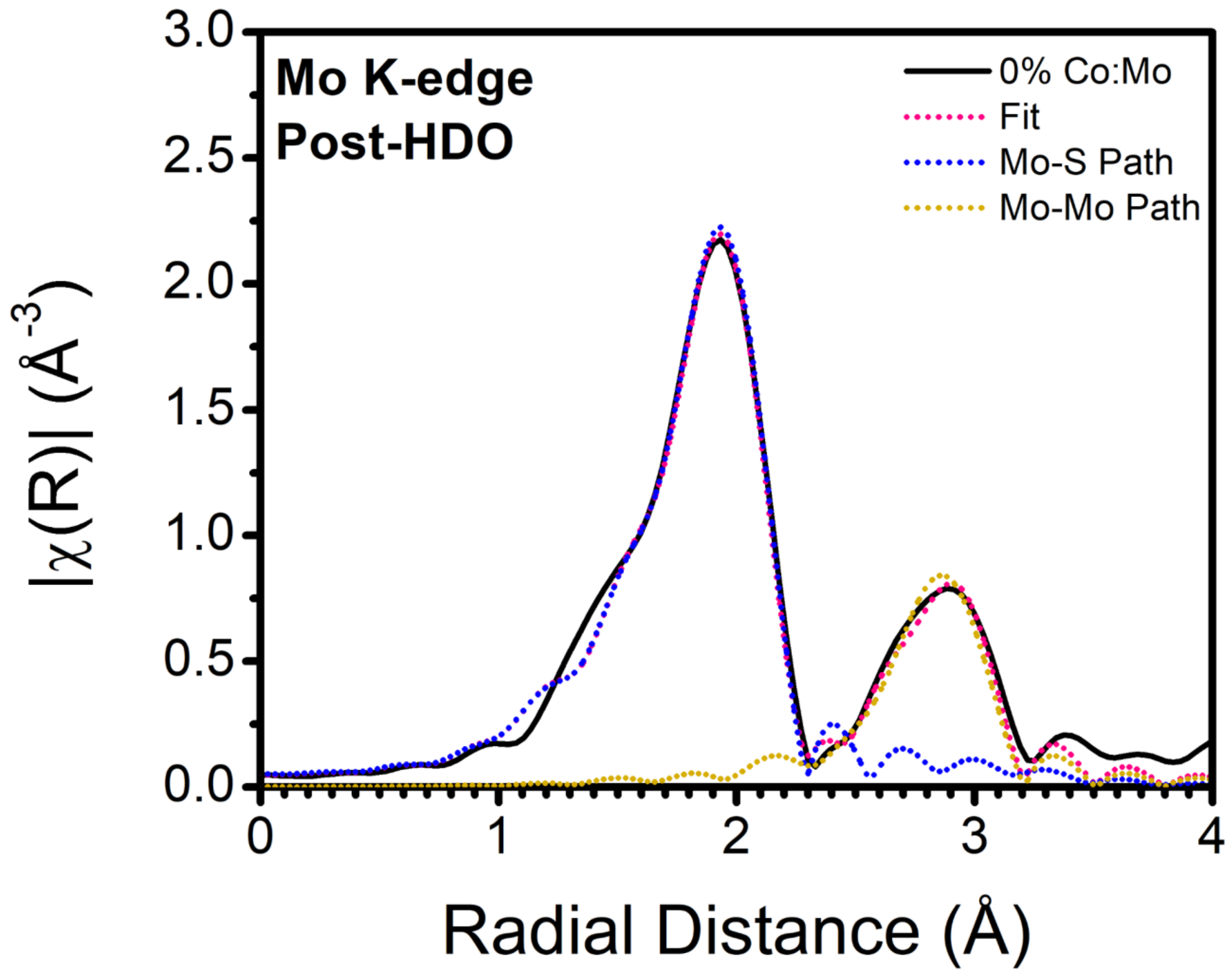

Figure S12. Fourier transform magnitudes of the k^2-weighted Mo K-edge EXAFS data and fits for the first- and second shells of 0% Co:Mo catalyst in Artemis, showing Mo-S and Mo-Mo path contributions.

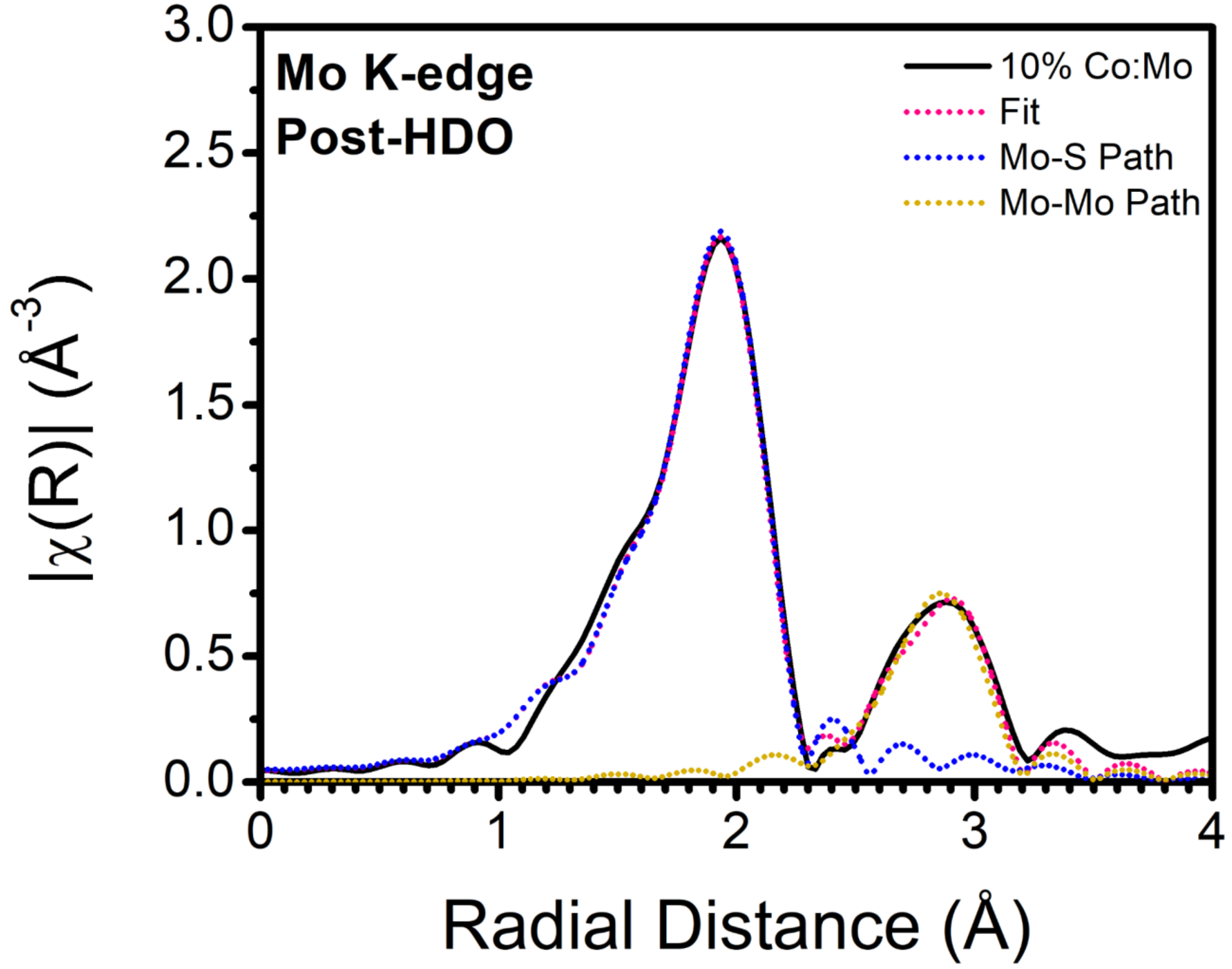

Figure S13. Fourier transform magnitudes of the k^2-weighted Mo K-edge EXAFS data and fits for the first- and second shells of 10% Co:Mo catalyst in Artemis, showing Mo-S and Mo-Mo path contributions.

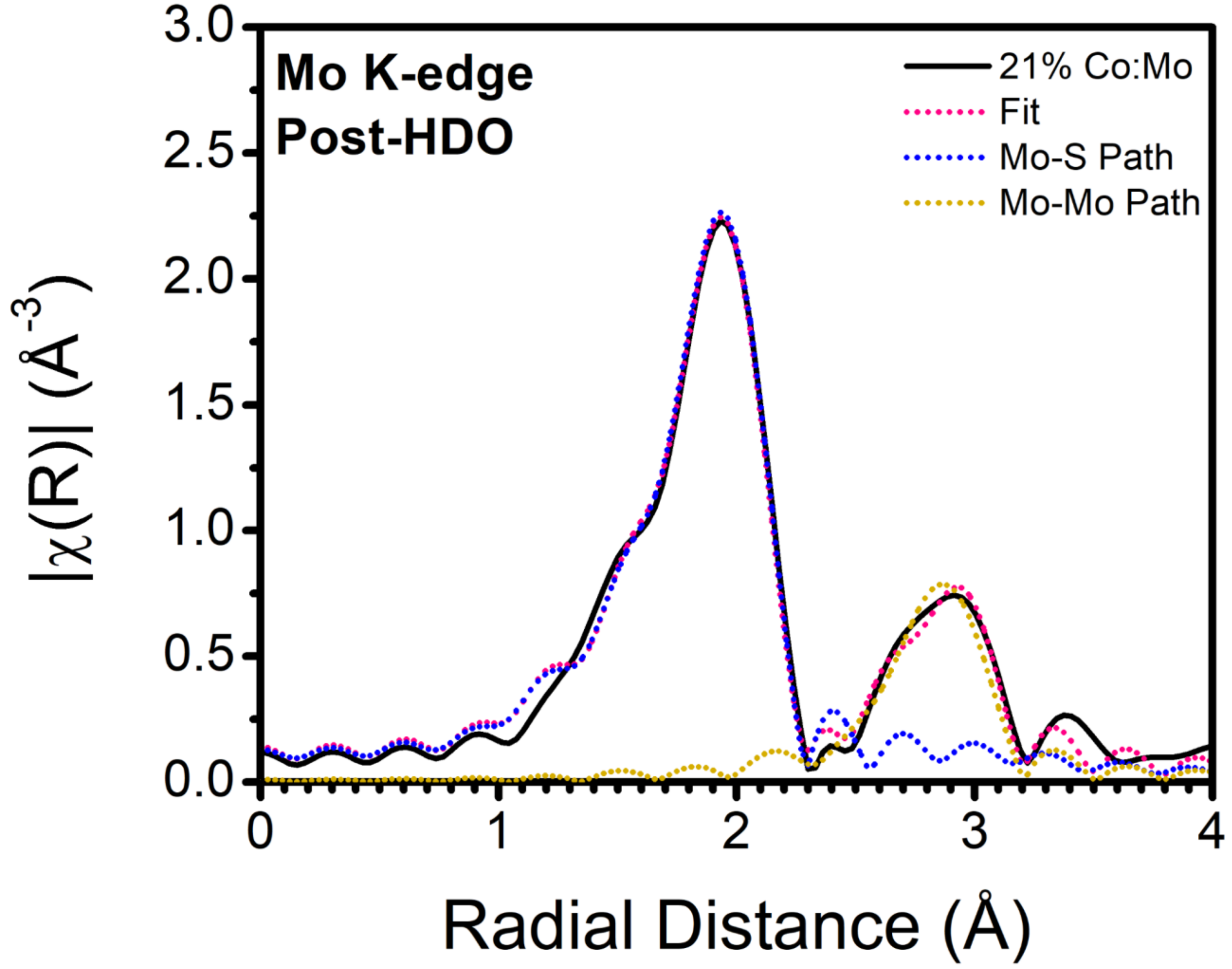

Figure S14 Fourier transform magnitudes of the k^2-weighted Mo K-edge EXAFS data and fits for the first- and second shells of 21% Co:Mo catalyst in Artemis, showing Mo-S and Mo-Mo path contributions.

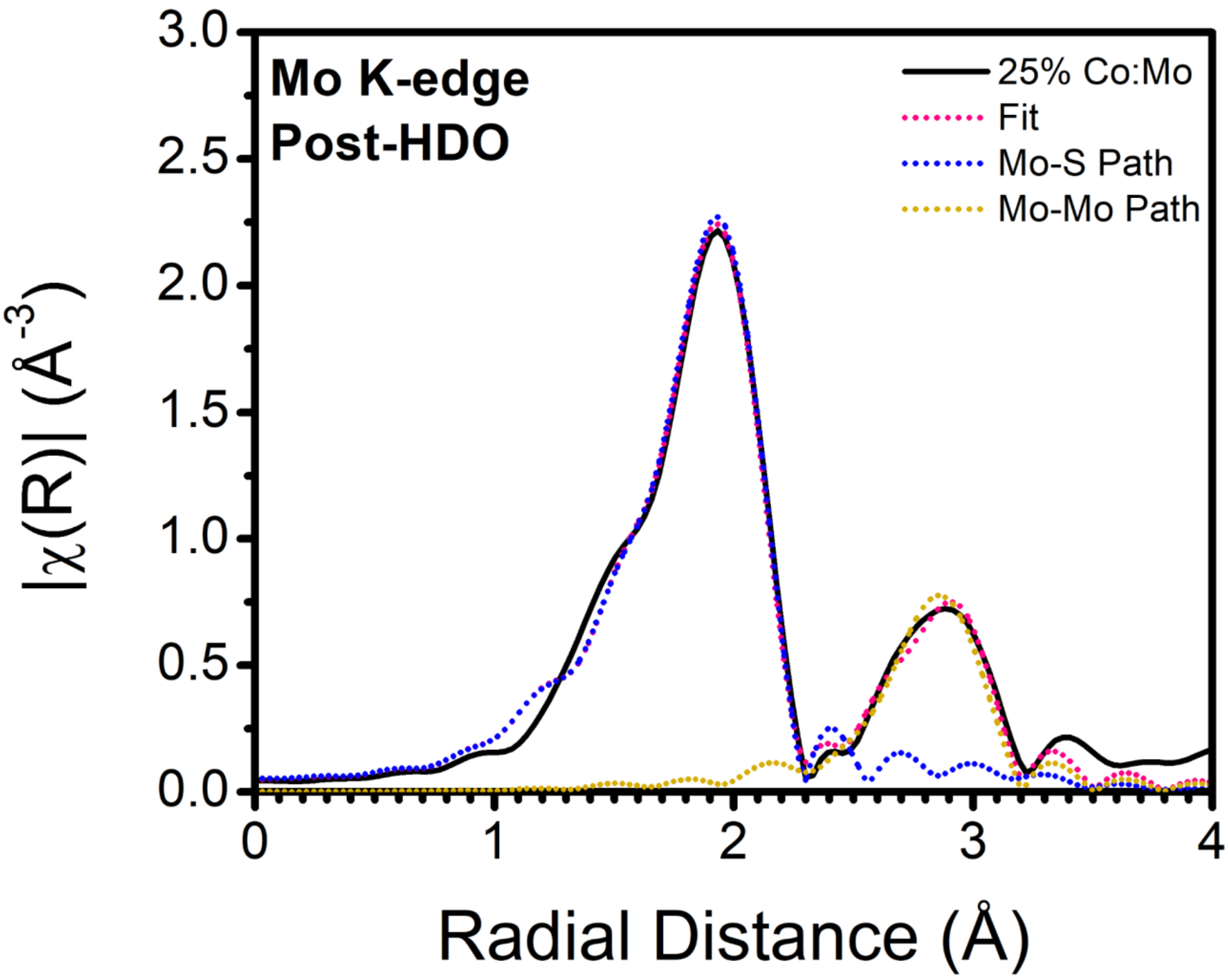

Figure S15. Fourier transform magnitudes of the k^2-weighted Mo K-edge EXAFS data and fits for the first- and second shells of 25% Co:Mo catalyst in Artemis, showing Mo-S and Mo-Mo path contributions.

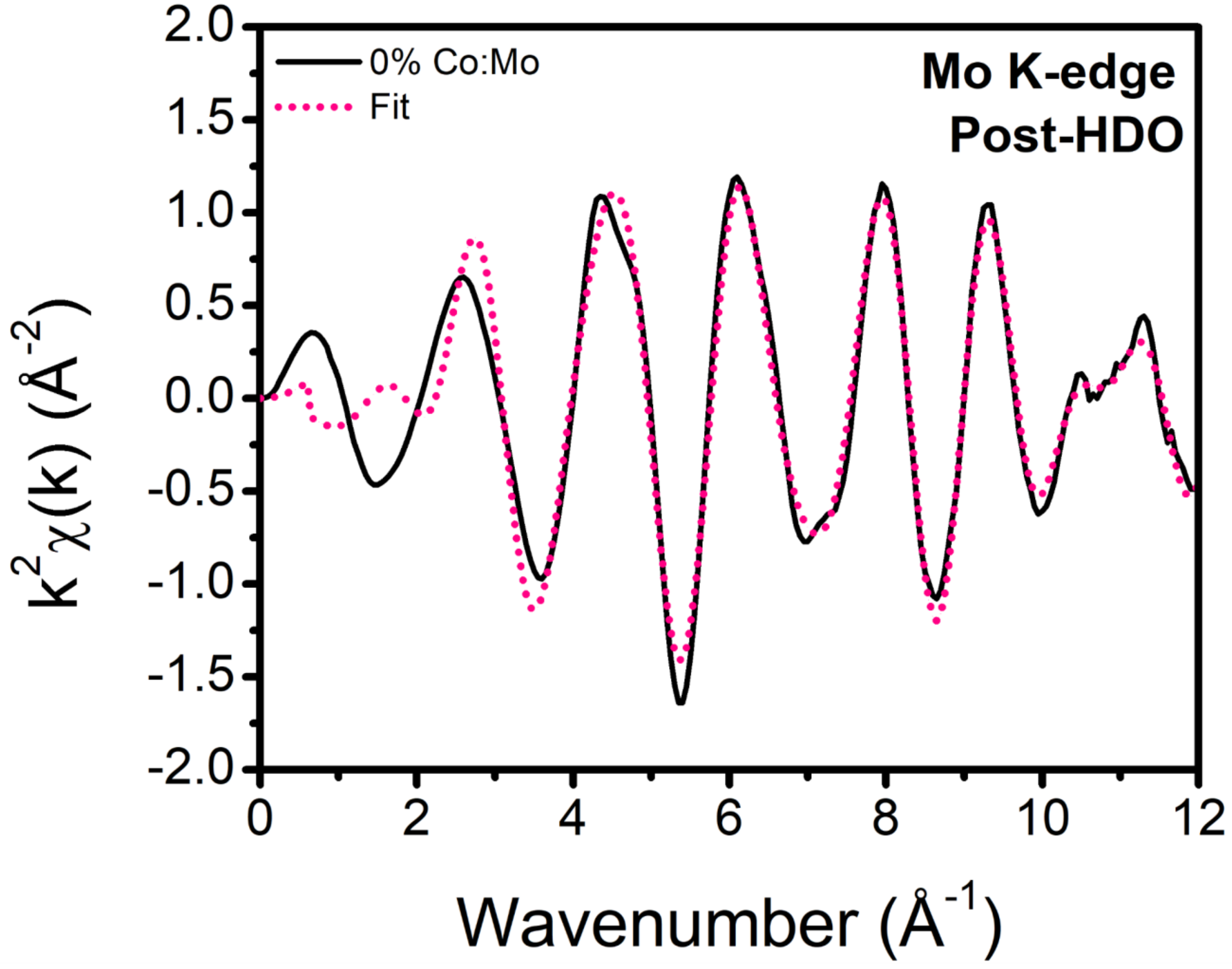

Figure S16. Mo K-edge k^2-weighted EXAFS data and fits for Mo K-edge on the 0% Co:Mo catalyst.

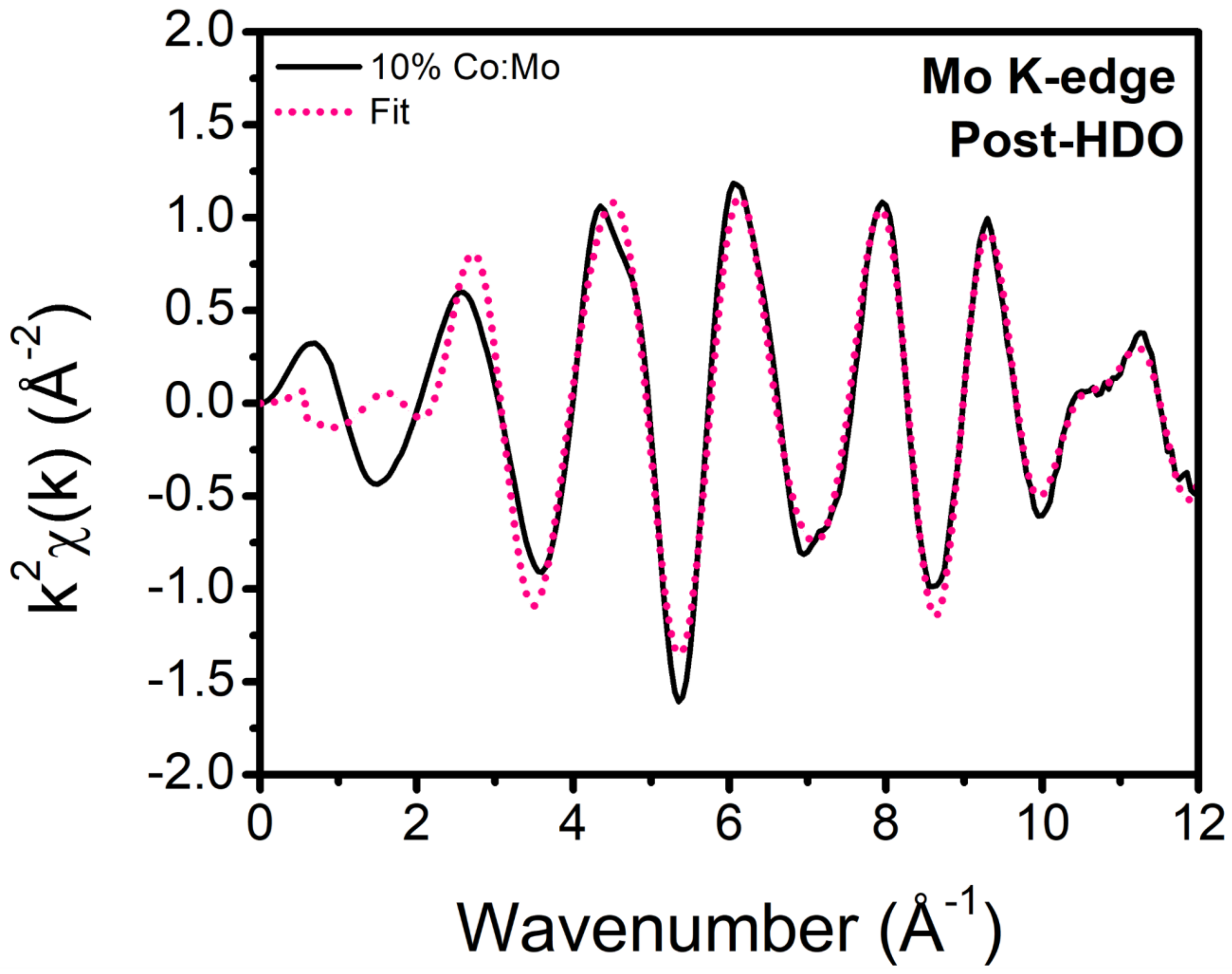

Figure S17. Mo K-edge k^2-weighted EXAFS data and fits for Mo K-edge on the 10% Co:Mo catalyst.

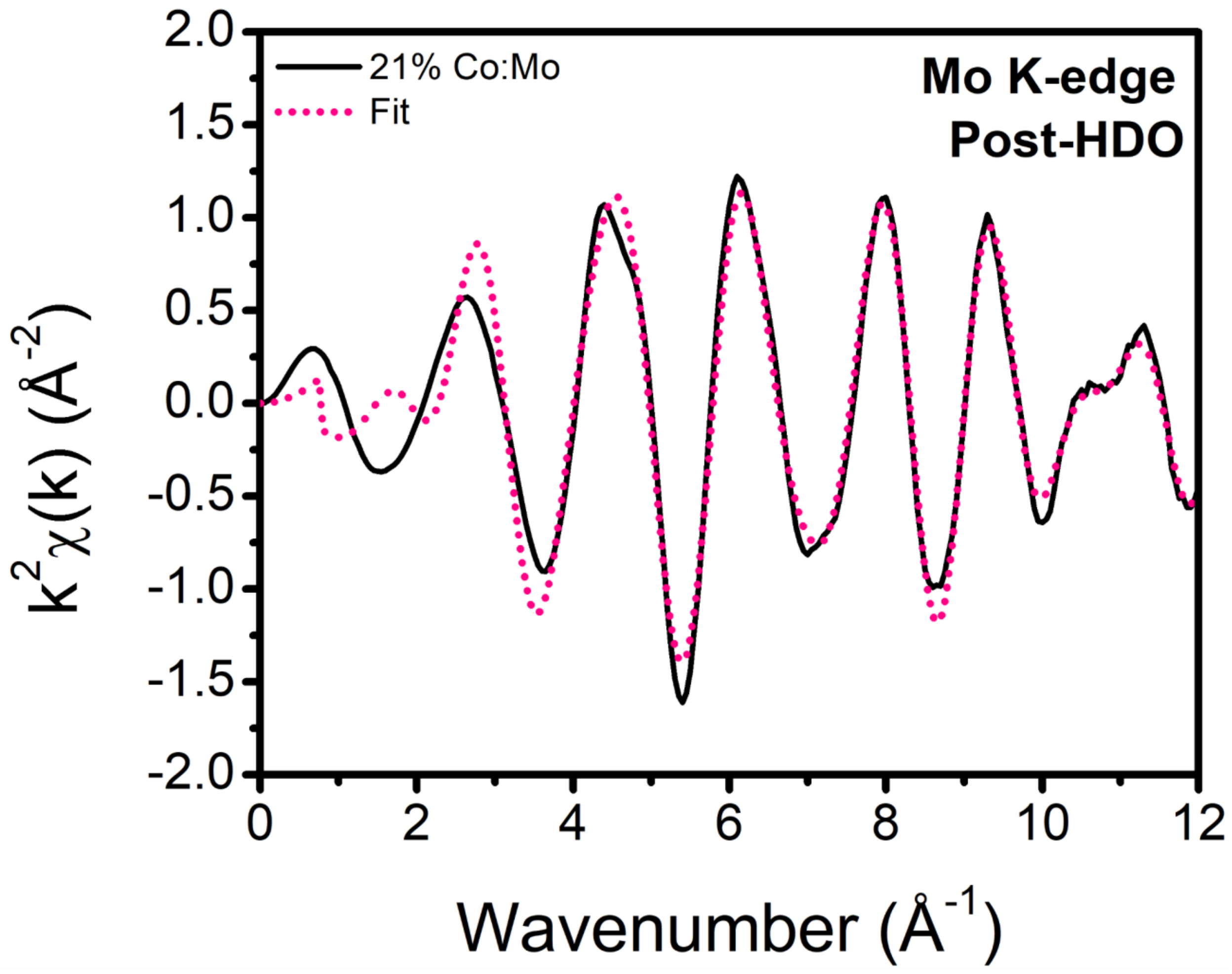

Figure S18. Mo K-edge k^2-weighted EXAFS data and fits for Mo K-edge on the 21% Co:Mo catalyst.

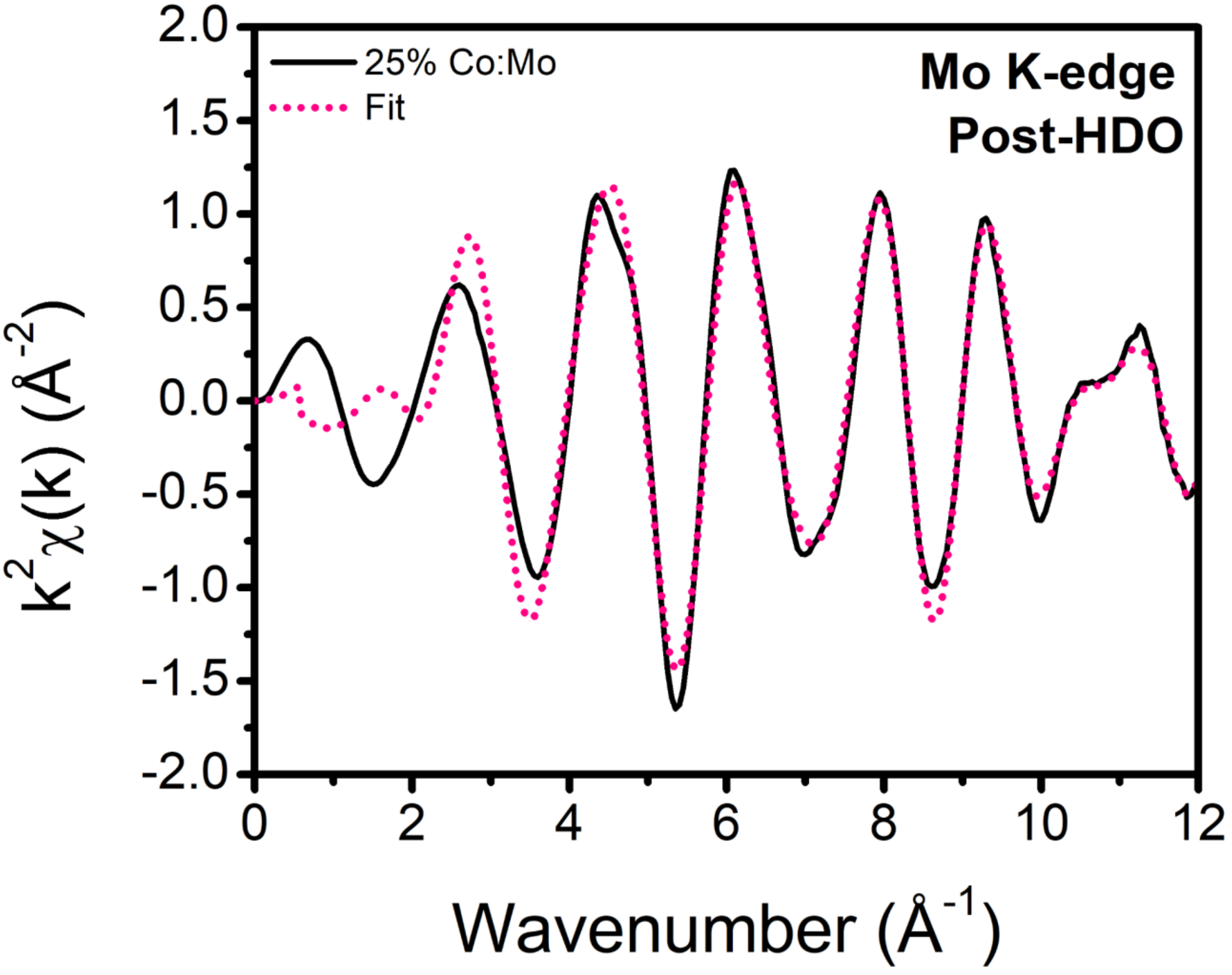

Figure S19. Mo K-edge k^2-weighted EXAFS data and fits for Mo K-edge on the 25% Co:Mo catalyst.

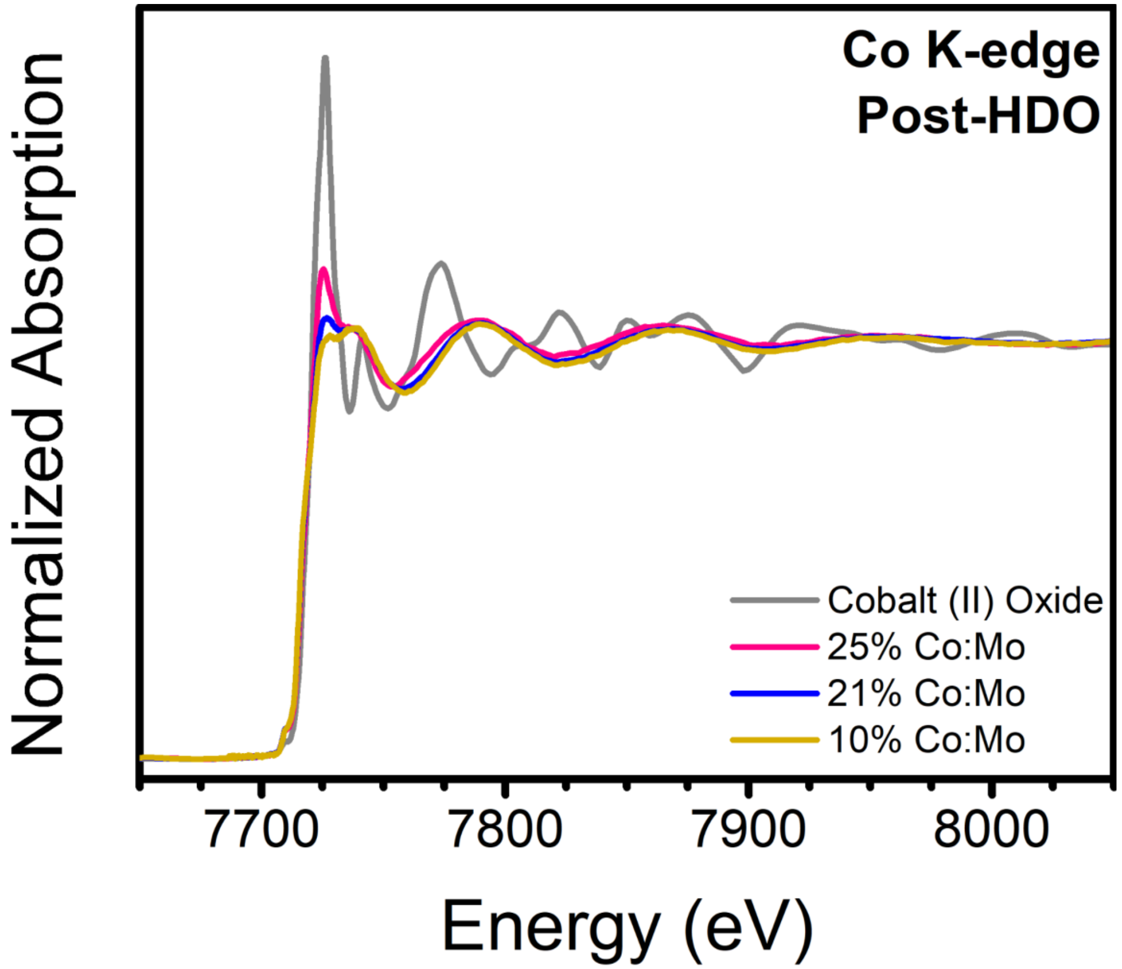

Figure S20. Co K-edge absorption coefficients in the XANES range for catalysts after 2 h under HDO conditions. The structures of the catalysts are generally similar, although we note the slight oxidation influence with increasing Co loading as evidenced by the increase in whiteline intensity appearing in 21% and 25% Co:Mo.

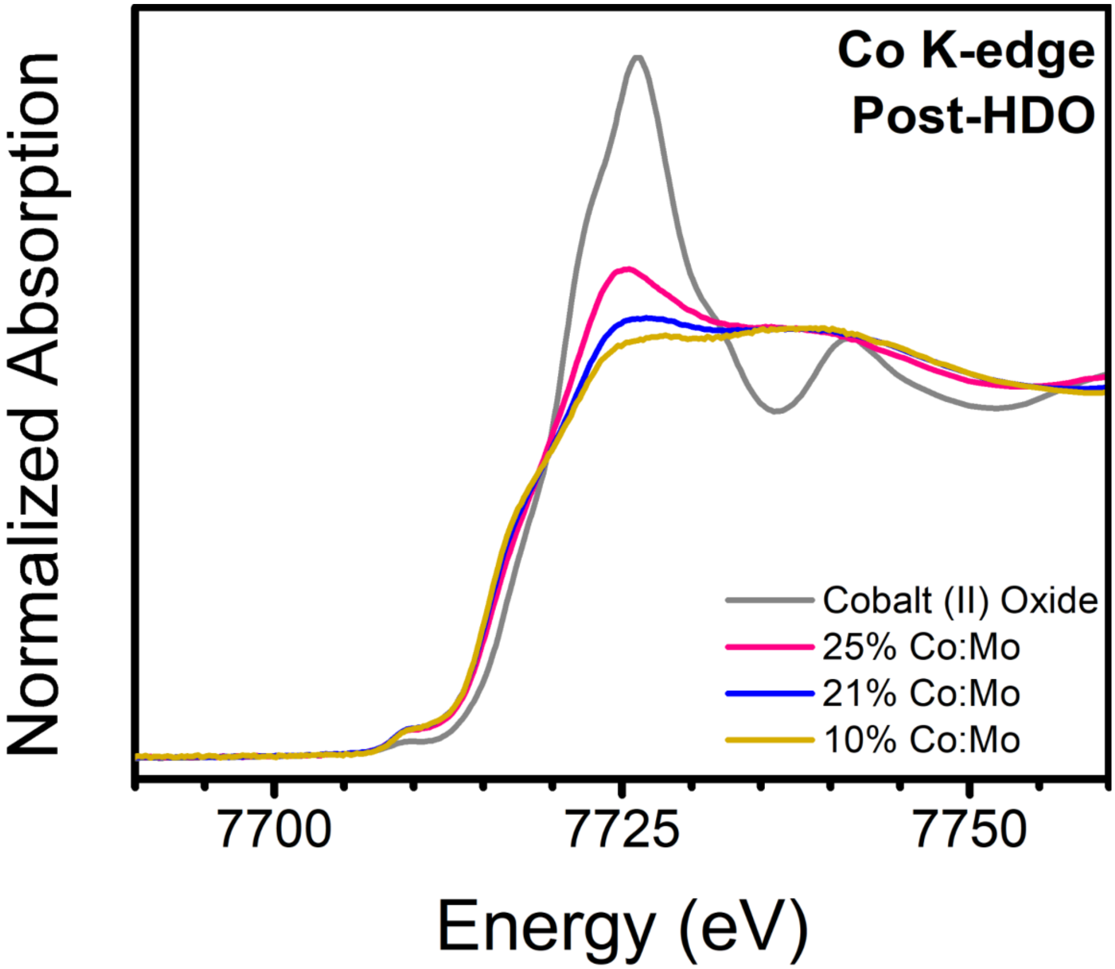

Figure S21. Co K-edge absorption coefficients in the XANES range for catalysts after 2 h under HDO conditions. There is a slight blue-shift of the absorption energy as Co loading increases, but far less than the cobalt (II) oxide reference. This further supports that the catalysts are primarily similar in structure and we are not forming cobalt oxide clusters on the surface.

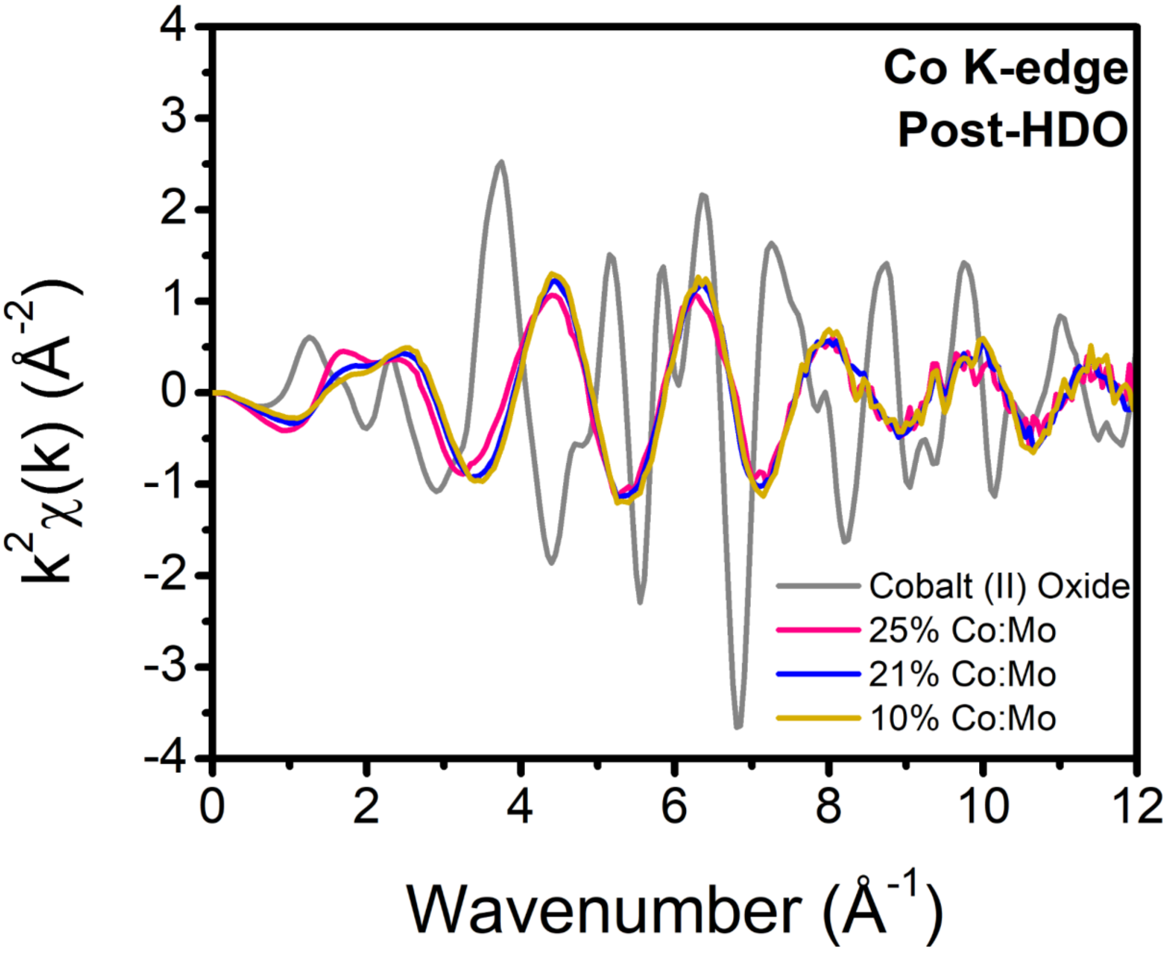

Figure S22. Co K-edge k^2-weighted EXAFS spectra in *k*-space of catalysts after 2 hrs under HDO conditions. The patterns of the catalysts are generally in phase and do not match well with the cobalt (II) oxide reference, even in samples we previously noted (21% and 25% Co:Mo) showed slight oxidation in XANES.

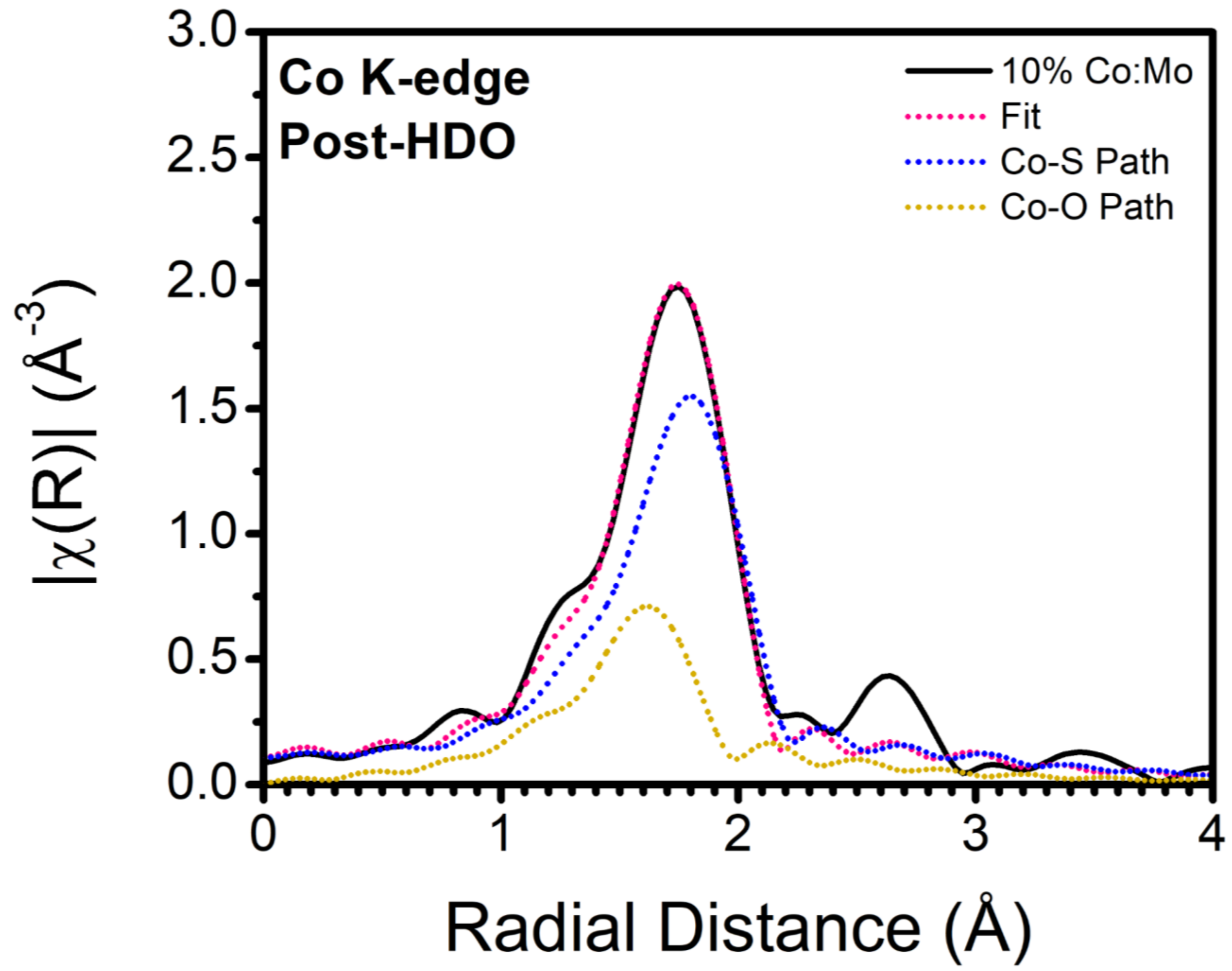

Figure S23. Fourier transform magnitudes of the k^2-weighted Co K-edge EXAFS data and fits for the first shell of 10% Co:Mo catalyst in Artemis, showing Co-S and Co-O path contributions.

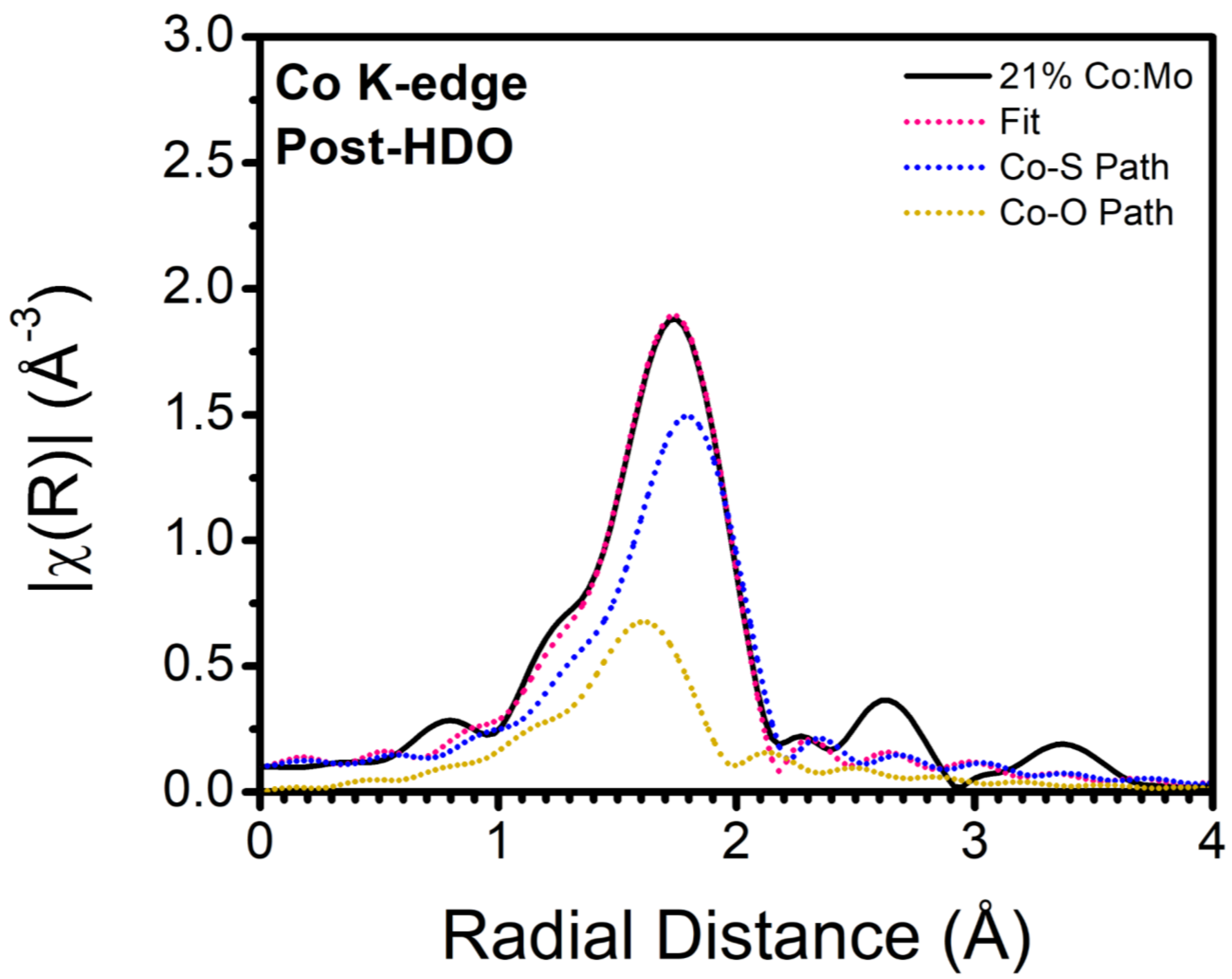

Figure S24. Fourier transform magnitudes of the k^2-weighted Co K-edge EXAFS data and fits for the first shell of 21% Co:Mo catalyst in Artemis, showing Co-S and Co-O path contributions.

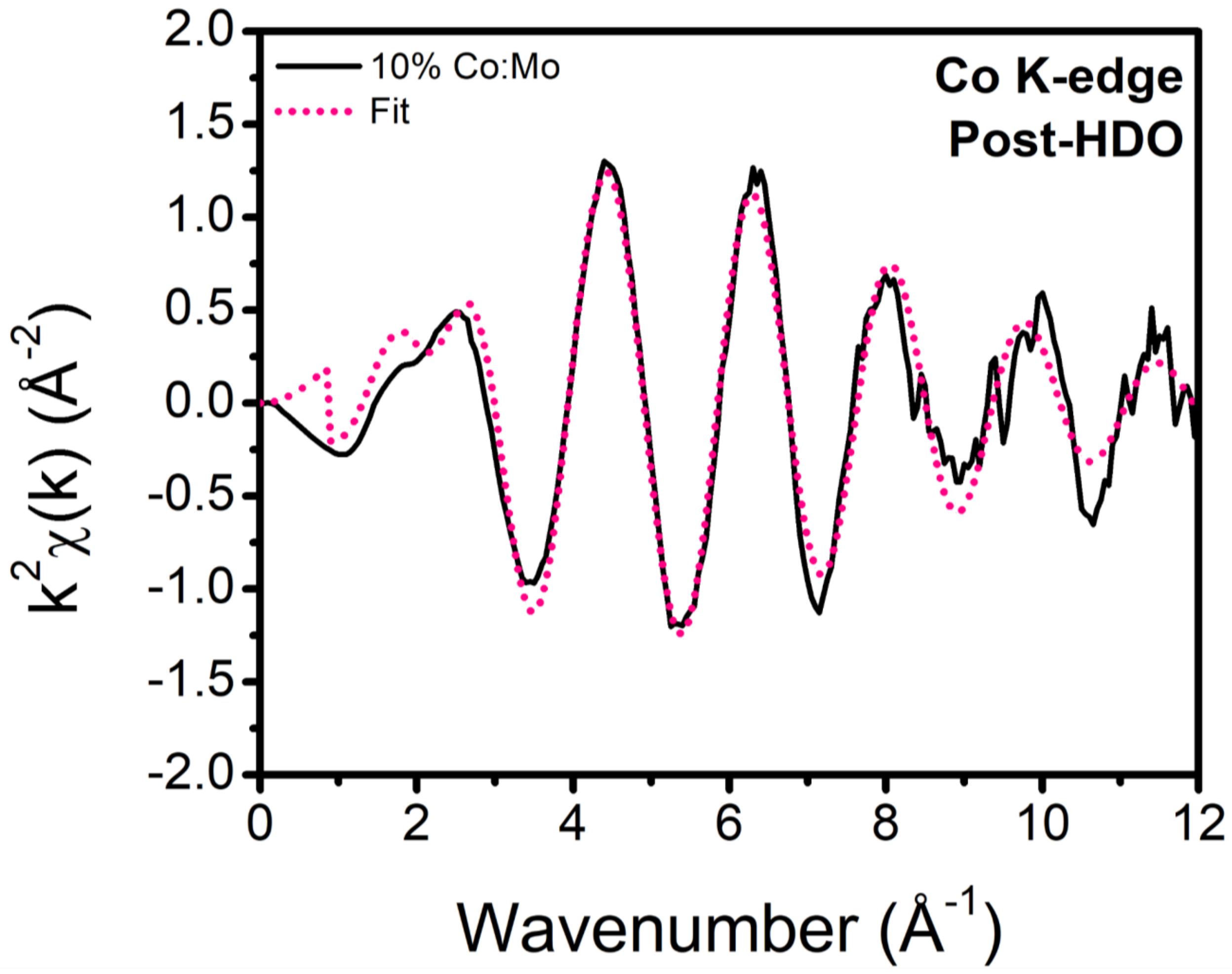

Figure S25. Co K-edge k^2-weighted EXAFS data and fits for Co K-edge on the 10% Co:Mo catalyst.

.

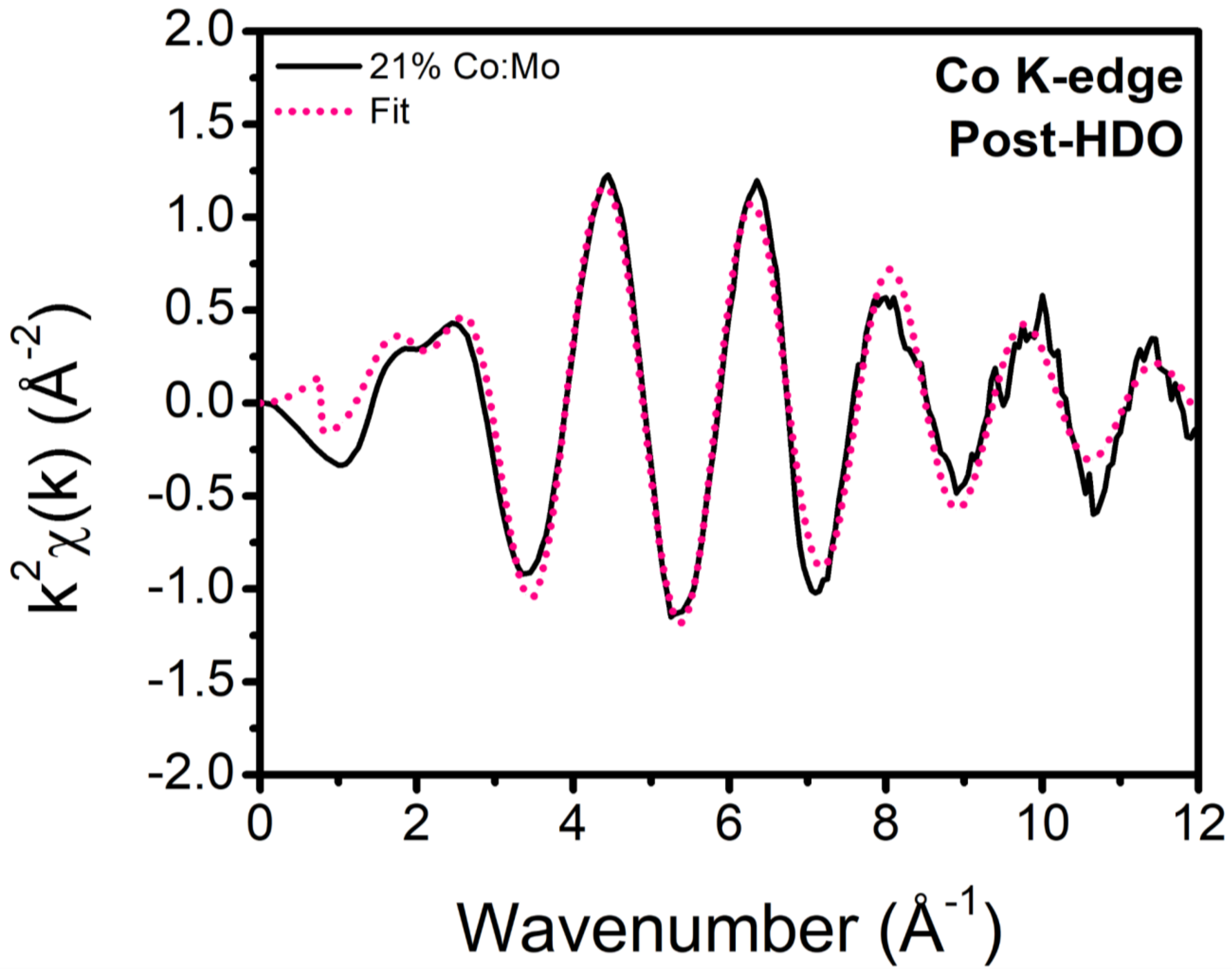

Figure S26. Co K-edge k^2-weighted EXAFS data and fits for Co K-edge on the 21% Co:Mo catalyst.

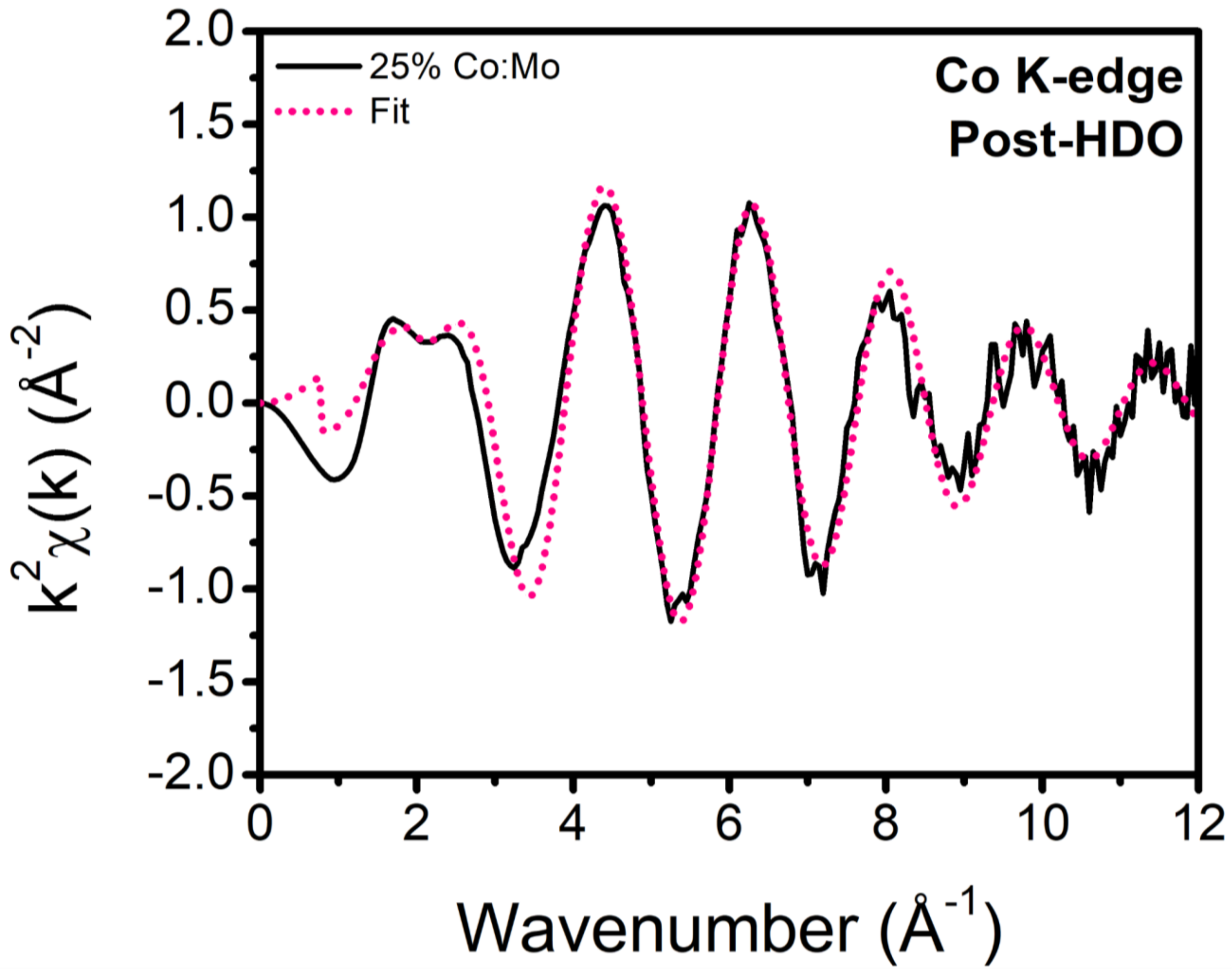

Figure S27. Co K-edge k^2-weighted EXAFS data and fits for Co K-edge on the 25% Co:Mo catalyst.

Table S1. EXAFS best fit parameters of the Mo K-edge in Artemis for the different Co:Mo catalysts post-HDO.

Co:Mo	Path	CN[a]	R (Å)	σ^2 (Å^2)	ΔE_0 (eV)	R-Factor
0%	Mo-S	6.0(3)	2.407(4)	0.0036(6)	1.6(6)	0.69%
	Mo-Mo	3.4(7)	3.162(5)	0.004(1)		
10%	Mo-S	5.8(3)	2.408(4)	0.0033(6)	1.2(6)	0.66%
	Mo-Mo	2.8(7)	3.164(6)	0.004(1)		
21%	Mo-S	5.9(3)	2.406(3)	0.0033(4)	2.4(5)	0.60%
	Mo-Mo	3.0(6)	3.163(4)	0.004(1)		
25%	Mo-S	6.3(3)	2.408(3)	0.0037(4)	1.4(5)	0.54%
	Mo-Mo	3.3(6)	3.162(4)	0.005(1)		

[a]Coordination Number

Table S2. EXAFS best fit parameters of the Co K-edge in Artemis for the different Co:Mo catalysts post-HDO.

Co:Mo	Path	CN	R (Å)	σ^2 (Å^2)	ΔE_0 (eV)	R-Factor
10%	Co-S	4.0(6)	2.23(1)	0.006(2)	3(2)	0.58%
	Co-O	2.0(4)	2.034(9)	0.002(2)		
21%	Co-S	3.7(5)	2.228(9)	0.006(1)	2(1)	0.37%
	Co-O	2.1(3)	2.034(7)	0.003(2)		
25%	Co-S	3.1(6)	2.22(1)	0.005(2)	0(2)	1.24%
	Co-O	2.7(7)	2.03(1)	0.008(5)		

Density Functional Theory Computations

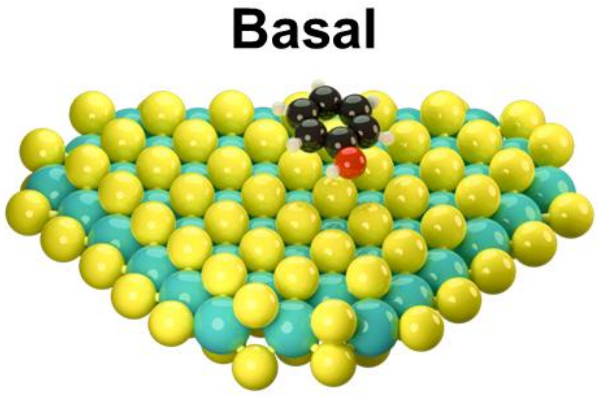

Figure S28. DFT modeled structures of phenol interacting with pristine MoS_2 nanosheets (undoped). The orientation of the phenol suggests that van der Waals forces drive interaction between the aromatic ring and the MoS_2 basal plane (-0.50 eV) and edge site (M5, -0.46 eV).

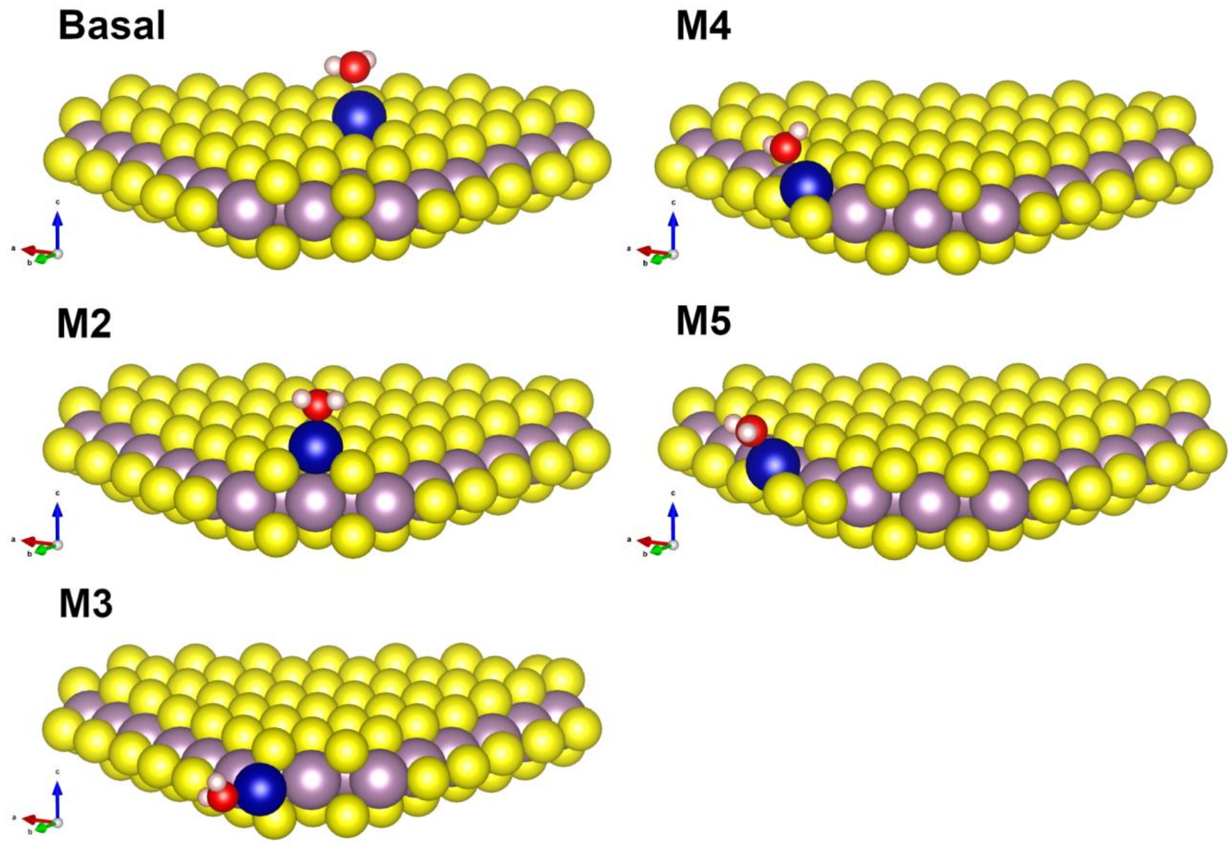

Figure S29. DFT modeled structures in VESTA depicting adsorption of H_2O on Co-MoS_2. Co, Mo, S, O, and H are depicted as blue, mauve, yellow, red, and white, respectively[3]

Table S3. Results and best fit parameters of EXAFS modeling at the Co K-edge for the different Co:Mo catalysts post-HDS. Results from Ref. 22 in main text.[4]

Co:Mo	Path	CN	R-fit (Å)	σ^2 (Å^2)	ΔE_0 (eV)	R-Factor
10%	Co-S	4.5(7)	2.221(7)	0.005(2)	0(2)	2.47%
	Co-O	0.6(6)	1.95(4)	0.000(9)		
21%	Co-S	3.9(3)	2.247(3)	0.0045(7)	5(1)	0.32%
	Co-O	1.9(2)	2.021(7)	0.003(2)		
25%	Co-S	3.8(5)	2.224(6)	0.006(1)	0(2)	1.50%
	Co-O	2.6(8)	2.04(2)	0.008(5)		
39%	Co-S	4.0(5)	2.206(5)	0.009(2)	-6(2)	0.93%
	Co-O	2.9(4)	2.047(6)	0.002(1)		

Table S4. Results and best fit parameters of EXAFS modeling at the Co K-edge for the different Co:Mo catalysts post-HDO.

Co:Mo	Path	CN	R-fit (Å)	σ^2 (Å^2)	ΔE_0 (eV)	R-Factor
10%	Co-S	4.0(6)	2.23(1)	0.006(2)	3(2)	0.58%
	Co-O	2.0(4)	2.034(9)	0.002(2)		
21%	Co-S	3.7(5)	2.228(9)	0.006(1)	2(1)	0.37%
	Co-O	2.1(3)	2.034(7)	0.003(2)		
25%	Co-S	3.1(6)	2.22(1)	0.005(2)	0(2)	1.24%
	Co-O	2.7(7)	2.03(1)	0.008(5)		

Calculation of Edge Site Saturation

Three geometries were considered for calculating the saturation point of Co doping on the edges. Each geometry was assumed to be 13 unit cells wide (unit cell = 3.19Å x 3.19Å in *a* and *b* dimensions). This equates to ~4.15nm wide nanosheets, close to the 4.21nm average diameter observed in HRTEM (see Figure 2b in main text).

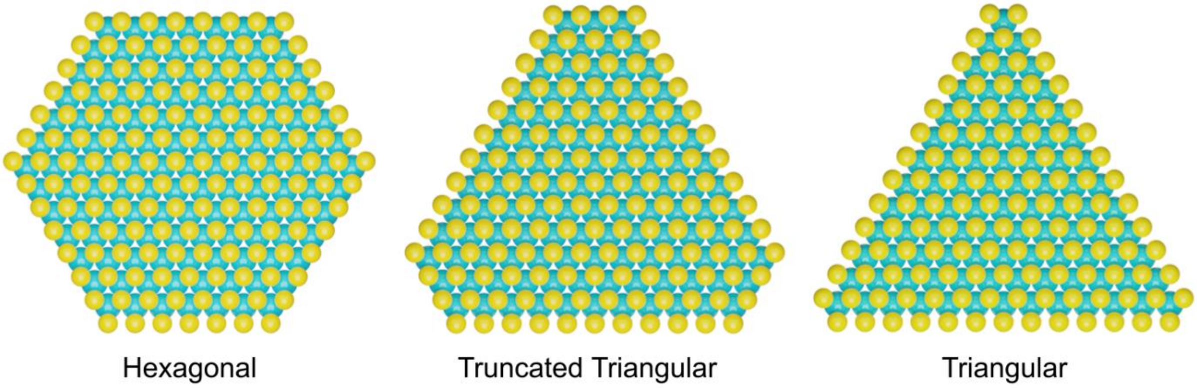

Figure S30. MoS_2 geometries considered for edge saturation calculations.

Hexagonal

A regular hexagonal morphology is assumed with 13 unit cells at its widest, with each side measuring 7 unit cells long. The sides alternate between S-edge and Mo-edge, although for the purposes of calculation we assume one Co can dope per edge unit cell regardless of the side. A hexagon represents the structure with the minimum ratio of edge sites to total Mo atoms.

Truncated Triangular

A hexagonal morphology is assumed, 13 unit cells at its widest. The three Mo-edge sides are assumed to be 11 unit cells long each, alternating with three S-edge sides that are 3 unit cells long each.

Triangular

An equilateral triangle morphology is assumed with 13 unit cells on each side. The sides are all assumed to be Mo-edge. An equilateral triangle represents the structure with the maximum ratio of edge sites to total Mo atoms.

Table S5. Calculation of atom and site counts for considered geometries of MoS_2.

Morphology	***Total Mo Atoms***	***Total Edge Sites***	***Edge Sites/ Total Mo Atoms***	***Co Saturation***
Hexagonal	127	36	28.3%	14.2%
Truncated Triangular	111	36	32.4%	16.2%
Triangular	91	36	39.6%	19.8%

Based on our observations, we assume our catalyst is predominantly a truncated triangle in shape. The Co saturation arises from the fact that Co cannot bind to every site due to steric hindrances (and no Co-Co nearest neighbors are observed in EXAFS), so saturation would occur by occupying half of all edge sites.

References

1. Ravel, B.; Newville, M. ATHENA, ARTEMIS, HEPHAESTUS: data analysis for X-ray absorption spectroscopy using *IFEFFIT*. *J. Synchrotron Radiat.* **2005**, *12* (4), 537-541. DOI: 10.1107/S0909049505012719.
2. Jain, A.; Ong, S. P.; Hautier, G.; Chen, W.; Richards, W. D.; Dacek, S.; Cholia, S.; Gunter, D.; Skinner, D.; Ceder, G.; Persson, K. A. Commentary: The Materials Project: A materials genome approach to accelerating materials innovation. *APL Mater.* **2013**, *1*, 011002. DOI: 10.1063/1.4812323.
3. Momma, K.; Izumi, F.; *VESTA 3* for three-dimensional visualization of crystal, volumetric and morphology data. *J. Appl. Cryst.* **2011**, *44*, 1272-1276. DOI: 10.1107/S0021889811038970.
4. Farrell, S. L.; Khwaja, M.; Paredes, I. J.; Oyuela, C.; Clarke, W.; Osinski, N.; Ebrahim, A. M.; Paul, S. J.; Kannan, H.; Mølnås, H.; Ma, L.; Ehrlich, S. N.; Liu, X.; Riedo, E.; Rangarajan, S.; Frenkel, A. I.; Sahu, A. Elucidating Local Structure and Positional Effect of Dopants in Colloidal Transition Metal Dichalcogenide Nanosheets for Catalytic Hydrogenolysis. *J. Phys. Chem. C* **2024**, *128* (11), 4470-4482.
